# Influence of T-Bar on Calcium Concentration Impacting Release Probability

**DOI:** 10.3389/fncom.2022.855746

**Published:** 2022-05-02

**Authors:** Markus M. Knodel, Ranjita Dutta Roy, Gabriel Wittum

**Affiliations:** ^1^Goethe Center for Scientific Computing (GCSC), Goethe Universität Frankfurt, Frankfurt, Germany; ^2^Science for Life Laboratory, KTH Stockholm, Stockholm, Sweden; ^3^Applied Mathematics and Computational Science, Computer, Electrical and Mathematical Science and Engineering Division, King Abdullah University of Science and Technology, Thuwal, Saudi Arabia

**Keywords:** T-bar, Drosophila larval NMJ, VGCC, calcium influx, diffusion-reaction PDE model, calcium microdomain, vesicle release probability, UG4

## Abstract

The relation of form and function, namely the impact of the synaptic anatomy on calcium dynamics in the presynaptic bouton, is a major challenge of present (computational) neuroscience at a cellular level. The Drosophila larval neuromuscular junction (NMJ) is a simple model system, which allows studying basic effects in a rather simple way. This synapse harbors several special structures. In particular, in opposite to standard vertebrate synapses, the presynaptic boutons are rather large, and they have several presynaptic zones. In these zones, different types of anatomical structures are present. Some of the zones bear a so-called T-bar, a particular anatomical structure. The geometric form of the T-bar resembles the shape of the letter “T” or a table with one leg. When an action potential arises, calcium influx is triggered. The probability of vesicle docking and neurotransmitter release is superlinearly proportional to the concentration of calcium close to the vesicular release site. It is tempting to assume that the T-bar causes some sort of calcium accumulation and hence triggers a higher release probability and thus enhances neurotransmitter exocytosis. In order to study this influence in a quantitative manner, we constructed a typical T-bar geometry and compared the calcium concentration close to the active zones (AZs). We compared the case of synapses with and without T-bars. Indeed, we found a substantial influence of the T-bar structure on the presynaptic calcium concentrations close to the AZs, indicating that this anatomical structure increases vesicle release probability. Therefore, our study reveals how the T-bar zone implies a strong relation between form and function. Our study answers the question of experimental studies (namely “Wichmann and Sigrist, Journal of neurogenetics 2010”) concerning the sense of the anatomical structure of the T-bar.

## 1. Introduction

### 1.1. Experimental Biological Basis and Questions

#### 1.1.1. Calcium Dynamics at Presynaptic Boutons

Understanding the plasticity of neuronal connections, neuronal activity, and the relation of form and function of neuronal anatomical structures is a major challenge of present day neuroscience.

Of special importance are the relation of calcium dynamics, anatomical shapes, and release probabilities of synaptic vesicles at AZs of neuronal synapses. For an extended overview we refer to e.g., Naraghi and Neher, 1997; Naraghi et al., 1998; Egelman and Montague, 1999; Koester and Sakmann, 2000; Kuromi and Kidokoro, 2000; Meinrenken et al., 2002, 2003; Erler et al., 2004; Jordan et al., 2005; Taschenberger et al., 2005; Gaffield et al., 2006; Steinert et al., 2006; Hosoi et al., 2007; Wölfel et al., 2007; Westphal et al., 2008; Denker et al., 2009; Frank et al., 2009; Pan and Zucker, 2009; Südhof, 2012; Wong et al., 2014; Goel et al., 2019b.

Here, we repeat in brief the major elements relevant as the basis for our study:

When an axon of a nerve is stimulated with an action potential, the elevated voltage causes calcium influx at the voltage-gated calcium channels of the AZs. This means that calcium is transported actively from the synaptic cleft into the presynaptic boutons. The calcium that enters the presynaptic boutons creates a calcium microdomain close to the release zone. Calcium microdomains trigger the fusion of presynaptic vesicles with the membrane *via* the calcium sensitive fusion sensor. After fusion, the vesicles exocytose neurotransmitters into the synaptic cleft. The neurotransmitter evokes postsynaptic potentials (EPSPs). In the case of a neuromuscular junction, the muscle contracts.

In more detail: after the calcium has entered the presynaptic zone, it experiences several processes that reduce its concentration again rather soon: It diffuses away inside the presynaptic boutons, it gets buffered with the aid of specific buffers such as calbindin, and calcium pumps pump it again into the synaptic cleft. Therefore, the calcium microdomain will be decreased soon again. Anyhow, the small but strong calcium influx has an impact upon the vesicles, especially for those who belong to the readily releasable pool, which presumably is located close to the AZ. Vesicle release probability is related to calcium concentration in presynaptic AZs. Higher calcium concentrations in the calcium microdomains, which arise due to action potential stimulation enhance vesicle release probability. At each action potential, the calcium concentration peak triggers vesicle release (Parsons et al., 1994).

In the case of many synapses, the presynaptic region close to the AZ can be considered to be more or less homogeneous besides the vesicles, which are located there.

However, many synapses show distinct morphological features such that the presynaptic AZ bears specific anatomic structures, which might have an influence upon calcium dynamics and profile (Chou et al., 2020). Such examples are synapses in the sensory pathway which bear so-called “ribbons” (Chakrabarti and Wichmann, 2019), or pyramidal dense projections showing a grid-like structure in the CNS of vertebrates (Phillips et al., 2001) with a wide range of structures (Lenzi and Von Gersdorff, 2001). Despite different structures, it is likely that all these anatomical nonisotropic arrangements are important to modulate the presynaptic mechanisms, namely to orchestrate the presynaptic vesicle cycle properties and exocytosis in different types of animals (Muresan et al., 1999; Harlow et al., 2001).

Hence, distinct morphological structures and features are important for the function of several synapse types of neuronal transmission.

#### 1.1.2. The T-Bar Anatomical Obstacle

Indeed, some synapses harbor a special structure called a T-bar (cf. e.g., the review Wichmann and Sigrist, 2010 and original literature such as Atwood et al., 1993; Jia et al., 1993; Meinertzhagen, 1996; Feeney et al., 1998; Reist et al., 1998; Koenig and Ikeda, 1999; Yasuyama et al., 2002; Kittel et al., 2006; Prokop and Meinertzhagen, 2006; Wagh et al., 2006; Takemura et al., 2008; Fouquet et al., 2009; Johnson et al., 2009; Nieratschker et al., 2009; Owald and Sigrist, 2009; Owald et al., 2010), which at first sight could be considered to be similar to the letter “T,” and in 3D images resembled at first to be similar to a table, with one inner table leg.

Thus, a prominent example of a geometric non-homogeneous anatomical structure within an AZ is the T-bar structure of the Drosophila neuromuscular junction (NMJ) (Jan and Jan, 1976; Atwood et al., 1993). As it has a “table-like” geometric shape, Wichmann and Sigrist (2010), it is tempting to assume that this structure might be e.g., an obstacle for calcium to diffuse away as fast as in cases when the T-bar is not present. Indeed, the Drosophila NMJ presynaptic boutons harbor different AZ types: There are AZs, which bear such a T-bar, and there are AZs without a T-bar. In the case of a T-bar, usually, the calcium channels (Leunga and Byerly, 1991; Catterall and Few, 2008) show a clustered structure close to the T-bar, whereas, in the case of the lack of a T-bar, the calcium channels seem to be more distributed and not clustered in the majority of cases.

Indeed, the Drosophila larval NMJ is a relatively simple model system of synaptic plasticity, as it is an NMJ with only glutamatergic synapses. It harbors only excitatory effects, and no inhibitory ones, so that action potential input and evoked postsynaptic potentials (EPSPs) can be directly related. Therefore, this NMJ often serves as the basis for studying the biophysical interplay, which appears in the presynaptic vesicle cycle, the calcium dynamics, and the postsynaptic glutamate receptors. Concerning literature in this context, we refer to e.g., Allbritton et al., 1992; Helmchen et al., 1997; Klingauf and Neher, 1997; Naraghi and Neher, 1997; Xu et al., 1997; Augustin et al., 1999; Zhen and Jin, 1999; Nägerl et al., 2000; Fernández-Chacón et al., 2001; Neher and Sakaba, 2001; Matveev et al., 2002; Meinrenken et al., 2002; Rosenmund et al., 2002; Sugita et al., 2002; Varoqueaux et al., 2002; Basu et al., 2005, 2007; Lou and Schneggenburger, 2005; Rasse et al., 2005; Kittel et al., 2006; Füger et al., 2007; Wadel et al., 2007; Schmid et al., 2008; Wang et al., 2008; Fouquet et al., 2009; Graf et al., 2009; Young and Neher, 2009; Matz et al., 2010; Shin et al., 2010; Eggermann et al., 2011; Han et al., 2011; Haucke et al., 2011; Kaeser, 2011; Liu et al., 2011; Ma et al., 2011; Sigrist and Schmitz., 2011; Andlauer and Sigrist, 2012; Jahn and Fasshauer, 2012; Owald et al., 2012; Sigrist and Sabatini., 2012; Südhof, 2012; Gupta et al., 2013, 2016; Lipstein, 2013; Maglione and Sigrist., 2013; Matkovic et al., 2013; Spangler et al., 2013; Imig et al., 2014; Walter et al., 2014; Acuna et al., 2015; Böhme and Sigrist, 2015; Chen et al., 2015; Keller, 2015; Muhammad et al., 2015; Nakamura, 2015; Schotten et al., 2015; Ullrich et al., 2015; Böhme et al., 2016; Petzoldt et al., 2016; Sigrist and Petzoldt., 2016; Reddy-Alla et al., 2017.

In the review, Wichmann and Sigrist (2010) asked major questions concerning the T-bar of the Drosophila NMJ. Namely, it raised the question of the mechanistic impact of the AZ anatomy upon the vesicle cycle of exocytosis and endocytosis. Furthermore, Wichmann and Sigrist (2010) recalled that already Erich Buchner suggested that the Drosophila NMJ might be a suitable model system to address the question of the relationship and influence of form upon function.

More recent experimental studies (Graf et al., 2009; Goel et al., 2019a) stimulated further knowledge concerning the T-bar and the active zone anatomy of the NMJ: While (Graf et al., 2009) revealed that BRP mutants lack a T-bar, it is so far not directly clear if this lack is in relation to the reduced calcium influx, defects in the clustering of calcium channels, and low release probability. In each case, there are a lot of differences in several parameters of single AZs such as size, anatomy, and intensity (Guerrero et al., 2005; Ehmann et al., 2014; Van Vactor and Sigrist., 2017; Akbergenova et al., 2018; Gratz et al., 2019). Moreover, Marrus and DiAntonio. (2004), Kittel et al. (2006), Graf et al. (2009), Peled and Isacoff. (2011), Melom et al. (2013), Akbergenova et al. (2018); Goel et al. (2019a), Gratz et al. (2019) showed that calcium channel clustering, calcium channel numbers, calcium influx, and release probability are correlated with the size of the AZ and the size of the T-bar. Besides these challenges, it would be interesting to understand more profoundly why in endophilin mutants, the number of AZs is two times higher, but synaptic strength is kept similar because the sizes of the AZs are smaller (Goel et al., 2019a), resulting in maintained synaptic strength due to adjusted global neurotransmitter exocytosis.

All these studies unveiled important aspects of the T-bar such as size, its impact upon calcium dynamics and vesicle dynamics, as already summarized by Wichmann and Sigrist (2010). In order to extend the answers to these questions concerning the relation of form and function of the T-bar, it is tempting to figure out in more detail the biological sense of the specific anatomical T-bar structure, namely its impact upon vesicle release probability, which in turn is governed by the calcium microdomain concentration in the presynaptic AZ which arises due to calcium influx under action potential stimulation of the presynaptic boutons. Therefore, this study will focus on the impact of the T-bar anatomic obstacle upon the calcium microdomain concentration at the presynaptic AZ.

### 1.2. Aim of This *in silico* Study

In this study, we consider the dynamics of calcium, which underlies vesicle dynamics. In detail, we model and simulate the calcium current dynamics and calcium microdomain concentration of one AZ under action potential stimulation of the NMJ. We consider the relation of form and function, which appear at single AZs of the presynaptic boutons of the Drosophila larval NMJ.

To unveil the role of the T-bar at the active zone, we need to compare the different AZ types of the presynaptic boutons of the Drosophila larval NMJ. In detail, at the presynaptic boutons, there are AZs, which in part bear a T-bar, while others do not bear a T-bar. Thus, the major aim of this study will be to unveil the relation between the T-bar anatomical obstacle, its presence and absence, and the impact of this specific structure upon the calcium concentration profile within the AZ, close to the membrane. Our aim is to compare the calcium dynamics for AZs with T-bar with AZs without T-bar.

To perform the comparison of AZs with T-bar and AZs without T-bar, in this study, we generate *in silico* “anatomical structures” (which we call geometries in modelers language) with and without a T-bar. We will establish a mathematical model of calcium influx and diffusion at the AZ to compare the calcium microdomain concentration shape and profile for the case of the presence of a T-bar and its absence.

In order to investigate the interplay of the shape of the AZ and the distribution of the calcium channels, we developed a typical 3D geometry of a synapse with a T-bar and compared the calcium concentrations close to the AZ for the case of geometry with a T-bar and clustered channels with an AZ without T-bar, but still clustered channels, and an active zone without T-bar and without clustering of the calcium channels.

We simulated calcium influx under repetitive action potential stimulation and compared the calcium concentrations for the different cases in a fully 3D spatio-temporal resolved manner. This indicates that we calculated the calcium concentrations within the AZ at single points of the 3D space to reveal exactly the calcium distribution not only averaged over some compartments but fully resolved in 3D space and time. We evaluate the calcium concentrations as if we would have a high resolution microscope, but just *in silico* rather than *in vitro* or *in vivo*.

The study we present here is a purely theoretical study, without any earlier unpublished experimental data input, but using a significant amount of biological model parameters taken from the literature. The major questions of our study are to unveil the influence of the T-bar anatomic obstacle and the clustering structure of the calcium channels upon the calcium microdomain in the presynaptic AZ. Our *in silico* model simulations presented in this study show that the AZ with a T-bar obstacle and clustered calcium channels has substantially higher calcium microdomain concentrations in comparison to an AZ without T-bar but still with clustered calcium channels. Moreover, if the active zone lacks the T-bar, and moreover the calcium channels are not clustered, the calcium microdomain concentration will be even much lower in comparison to both aforementioned cases, an AZ with or without T-bar, but clustered calcium channels. We conclude that both, the T-bar obstacle and also the clustering structure of the calcium channels, have a major influence upon the calcium microdomain concentration and, thus, are major players of calcium plasticity of the NMJ in the sense that the presence or absence of the T-bar strongly influences calcium microdomain concentration and, thus, vesicle release probability.

This means that the T-bar obstacle has a substantial impact upon calcium microdomain concentration and shape, and this indicates that the T-bar anatomical structure likely has an important role to enhance vesicle release probability, as the concentration of calcium at the vesicular release sites is a major trigger of synaptic activity. Various biophysical models and simulations deal with the dynamics of this calcium in the context of synaptic activity, such as Naraghi and Neher (1997), Naraghi et al. (1998), Koester and Sakmann (2000), Meinrenken et al. (2002), Meinrenken et al. (2003), Erler et al. (2004), Taschenberger et al. (2005), Hosoi et al. (2007), Wölfel et al. (2007), Frank et al. (2009), and Wong et al. (2014).

The T-bar AZ likely is an optimal object to study the relation of form and function and their interplay within synaptic processes such as calcium dynamics and vesicle release probability.

Indeed, there exist several studies which considered the influence of an obstacle for calcium dependent vesicle exocystosis (cf. e.g., Kits et al., 1999; Segura J, 2000; Glavinovic and Rabie, 2001; Shahrezaei and Delaney, 2004; Biess A, 2011; Graydon et al., 2011).

Hence, there is ongoing modeling and simulation research to unravel the role of calcium at release sites, and it is promising to apply modeling and simulation techniques to the calcium dynamics processes appearing at the Drosophila NMJ synapses.

### 1.3. Organization of This Article

In Section 2, Materials and Models, we introduce the geometries representing typical anatomical scenarios for the synapses under consideration. The meshes created for these geometries will serve as the basis for the simulations. In particular, we introduce the mathematical model of calcium diffusion, reaction, and influx we use to describe calcium dynamics at the T-bar zone of the Drosophila NMJ larval synaptic AZ. In Section 3, Results, we present the simulation results of calcium influx and evaluation of calcium concentrations at the different geometric scenarios for the presynaptic AZ of the Drosophila larval NMJ boutons. In particular, we present the calcium concentration profiles in a spatially resolved manner and show how the calcium concentration behaves in the different scenarios. We can clearly relate the T-bar geometric obstacle with a quantitatively clearly enhanced calcium concentration at the AZ. In Section 4, Discussion, we interpret the simulation results and relate them to unravel the relation of form and function of the T-bar at the presynaptic AZ of the Drosophila larval NMJ boutons. In Section 5, Conclusion, we put our study in the broader context of present scientific research in the field of the interplay of calcium and vesicle dynamics at synaptic zones.

## 2. Materials and Models

**Figure 1:**
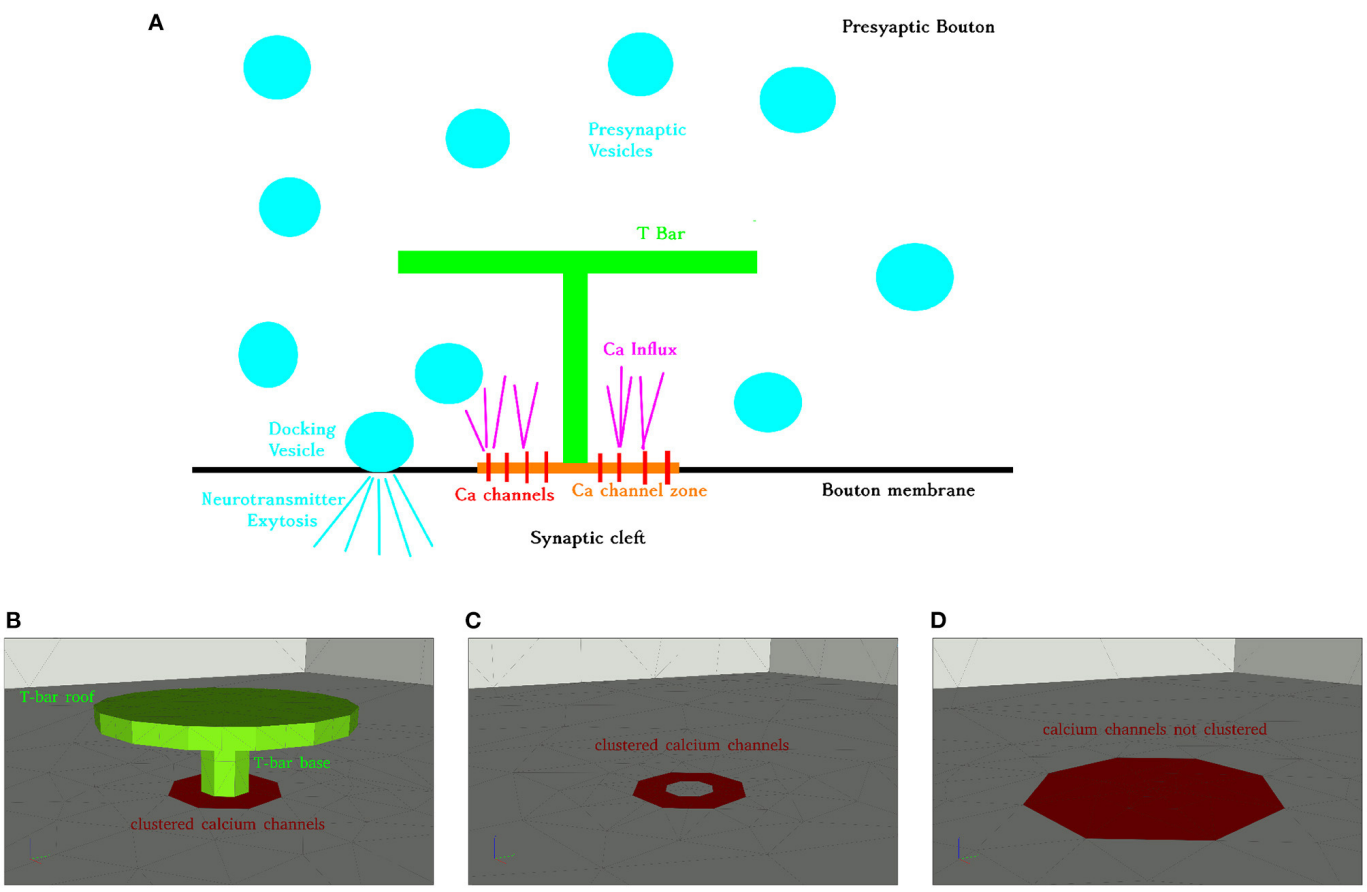
Aim of this figure: Display the biological process under consideration, and display the realization of the geometry of the anatomic structures for the three different anatomic structures under consideration. **(A)** Calcium influx and vesicle release at the Drosophila NMJ at an AZ with T-bar, schematic representation. **(B–D)**: Geometric realization of typical AZs of the NMJ: We consider three basic scenarios: **(A)** Active zone with T-bar and clustered channels, **(B)** AZ without T-bar, but with clustered channels, **(C)** AZ without T-bar, and the channels are not clustered, but more widely distributed (the surface where the channels are located is bigger, but the channel number is assumed to be the same). Reason for the difference between **(C,D)**: we want to probe for the influence of clustered channels in the case when no T-bar is present at the AZ. Therefore, the (red) channel zone is larger, and this reflects a biophysical change in the geometric conditions of the model. Note that the channel zones are marked with red color, and the T-bar with green color, i.e., channel zones C depicted with red color. Same perspective for all three cases. Scale: Diameter “table” of T-bar: 140 nm. Height of the leg: 40 nm, diameter of the leg: 30 nm. The thickness of the “table desk”: 10 nm. Diameter of the clustered channels: 70 nm, in case of not clustered channels: 140 nm.

To motivate the construction of the anatomy we use as the basis for our simulations, we re-consider the basic process of calcium influx, calcium microdomains, and vesicles in the AZ, cf. Figure 1A: Synaptic activity depends on the influx of action potentials. Once an electric action potential arrives at the AZ, extracellular calcium is injected at the voltage-gated calcium channels (located at the membrane between the presynaptic bouton and the synaptic cleft, at the site of the AZ) into the presynaptic AZ.

We made a mathematical model describing calcium dynamics in three dimensions in space plus in time, and simulated the influx of calcium caused by repetitive action potential stimulation. In particular, we modeled the intracellular calcium concentration by means of a diffusion-reaction model.

As N-type calcium channels are the major voltage-gated calcium channel type at synaptic terminals (Weber et al., 2010), we used the model published in Borg-Graham (1998) for voltage-gated calcium current (VGCC) (Weber et al., 2010) of N-type channels to describe the influx of calcium from the synaptic cleft into the presynaptic AZ. Calcium buffering, leakage, and binding is incorporated into our model as well. We studied the concentration of calcium close to the calcium channels with and without the T-bar.

### 2.1. Anatomical Structures and Finite Element Geometries

#### 2.1.1. Anatomical Structures - Surface Grids

Ultrastructural analysis of electron microscope images of the AZs at the presynaptic zones of the presynaptic boutons of the Drosophila NMJ revealed anatomical structures resembling “T-shaped structures” (Shaw and Meinertzhagen, 1986; Meinertzhagen, 1996; Wichmann and Sigrist, 2010). These structures show a “base”/“pedestal,” and a “roof”/“platform.” Therefore, these structures were called “T-bars,” Wichmann and Sigrist (2010).

To facilitate the naming of “roof” and “base” for readers, which have their origin more in the simulation community, we will use the more figurative words “table socket” and “table desk” in a synonymous way.

Using our mesh creation tool ProMesh4 (Reiter, 2014; Reiter et al., 2020), we constructed typical 3D geometries (surface grids), where the T-bar was either present or absent, (cf Figures 1B–D). The channels are integrated at the membrane which forms the base of the geometric zone. We assume the diameter of the round “table desk” to be 140 nm, the height of the leg to be 40 nm, the diameter of the leg to be 30 nm, the thickness of the “table desk” to be 10 nm. Our geometric assumptions of the T-bar geometry are based upon the EM images of Figure 6A of Sigrist et al. (2003) and Figure 6A of Sigrist et al. (2002). These figures display T-bars in EM images, including the size of the magnitude bar. Hence, the sizes of our 3D geometry construct representing the T-bar are typical, but not reconstructed. Nevertheless, we assume that they are sufficient to get *in silico* based insight into the relation of form and function. In the case of clustered channels, we assume a diameter of 70 nm for the VGCC region (assuming that the radius of the clustered region is half of the radius of the T-bar roof), and in case of not clustered channels, we assume a diameter of 140 nm for the VGCC region, i.e., of the same size as the table/roof of the T-bar (which in this case is not present).

The calcium channels are distributed in a continuous manner allover the 2D influx zone C located at the membrane, around the T-bar socket (the virtual T-bar socket in case when the T-bar is absent), i.e. around the center of the membrane at the AZ. We do not resolve single channels for our computations.

#### 2.1.2. Computational Mesh

As the calcium concentration far away from the AZ can be assumed to be small, as there is no influx, we may consider a region big enough around the T-bar AZ for our computations as the region of interest, where the simulations have to be performed, as the rest of the inner presynaptic zone will have negligible influence on the calcium concentrations, so we do not need to consider complete presynaptic boutons, in contrast to our former study about the relation of form and function of the presynaptic boutons (Knodel et al., 2014). Therefore, the triangular surface meshes displayed in Figure 1 (of T-bar, channel zone and membrane) are enclosed by a rectangular cuboid of size 1.2 × 1.2 × 0.6 μm and meshed by means of a tetrahedral volume mesh. The membrane coincides with the ground plate of the cuboid.

**Figure 2:**
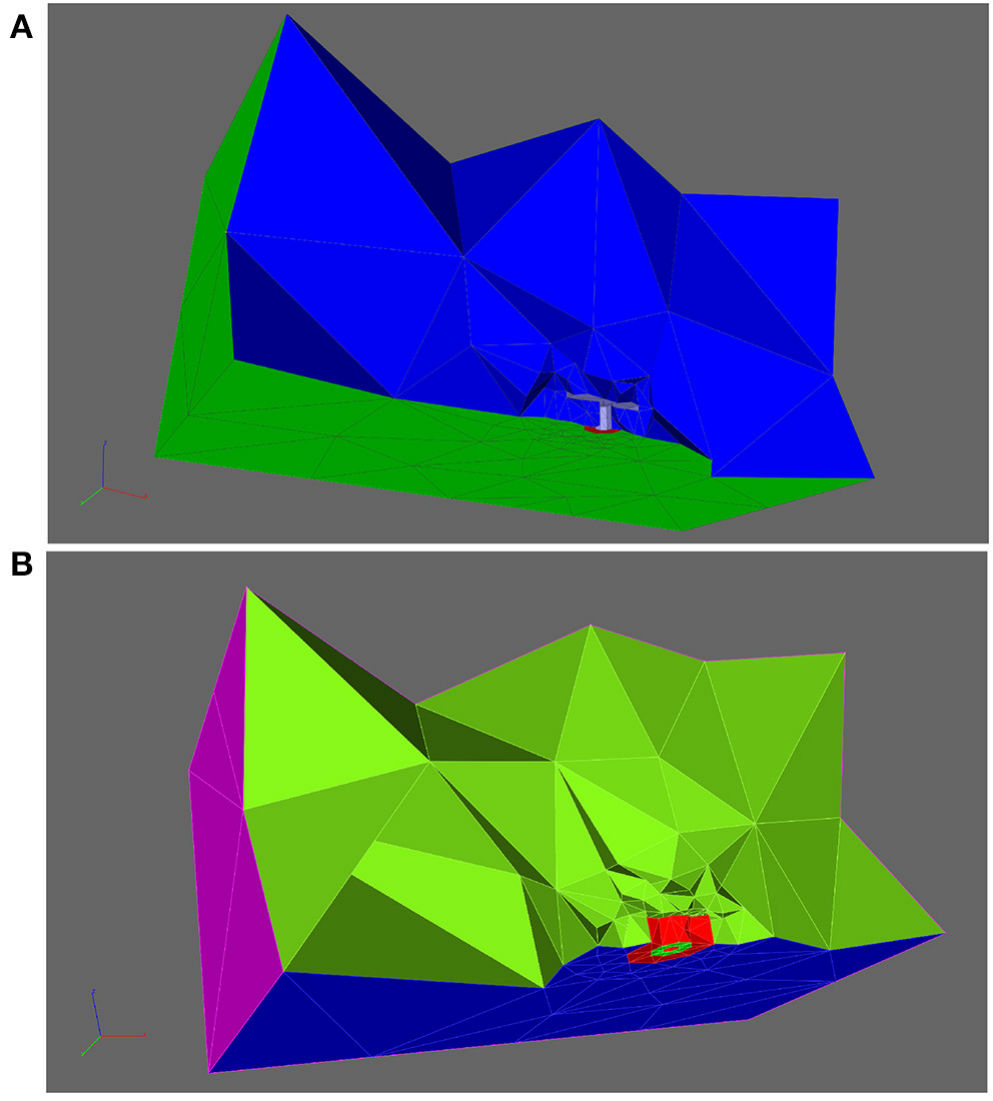
Aim of this figure: Display the computational domain in order to explain the geometric Finite Element mesh basis of the simulation. Computational domain displayed opened by a cut plane to allow insight into the region where the T-bar AZ is located, and to explain the subdomains which are important to understand the regions where computations are evaluated in this study. **(A)**: Cut of the volume mesh in case of the T-bar geometry, T-bar space left empty to account for the obstacle of the T-bar. Coarse grid. Entire computations are performed with refined grid versions. The complete 3D triangular mesh forms the computational domain V, blue. The surface zone of calcium channels, C, is marked in red. **(B)**: Cut plane applied to the volume mesh, where that volume “below” the “table” of the T-bar is highlighted with red color, the subdomain U. Note that the different colors compared to **(A)** do not indicate a paradigm change or other subdomains, but are applied in order to highlight the domain of interest we want to highlight here. Note also that the cut plane is located more in the front in comparison to the case of **(A)**.

In the case of the T-bar, the T-bar space is left blank in order to account for the obstacle of the T-bar, in the other case, the full space is meshed, in the case of the T-bar, we displayed a cut through the coarse form of the volume mesh, (cf. Figure 2).

The computational domain is denoted as V, i.e., the space filled with the 3D tetrahedral Finite Element mesh. The surface of the complete computational domain is denoted as S. The channel zone is denoted as C⊂S. The bottom of the cuboid except for the channel region is named B⊂S, and the side and top walls are called W⊂S. The surface of the T-bar socket and table is called T⊂S. T is the surface of that space which separates the T-bar “inner volume” (in the geometric sense) from the rest of the cuboid. This means that T separates the region inside the presynaptic bouton where calcium can diffuse from the anatomical T-bar obstacle. The T-bar “inner volume” is excluded from the computational domain and thus does not get a volume mesh. This strategy ensures that the simulated calcium cannot enter the T-bar obstacle.

Figure 2 depicts the volume mesh in a in which the part of the volume “below” the “table,” below the T-bar, is highlighted with a special color, the volume region U, where the calcium concentration might be enhanced due to the geometric obstacle.

### 2.2. Mathematical Partial Differential Equation (PDE) Model for Intracellular Calcium Dynamics

We model calcium influx and ion distribution by means of a diffusion-reaction 3D PDE model. Note that the use of a continuum approach to calculate calcium ion distribution has to be interpreted as a probability to find an ion in a certain location, similar to e.g., the Schrödinger equation in quantum mechanics to find an electron. In principle, the number of calcium (Ca^2+^) ions under the T-bar table is comparably few. The use of a continuum model, in fact, resembles the averaging of a huge number of computations performed e.g., with random walk algorithms. If we would perform a single computation with e.g., random walk models, the results would have to be averaged anyhow at the end. The use of a continuum model in the sense of a distribution probability enables to homogenize the model and to perform much fewer computations to arrive at the same biophysical result. These homogenization techniques are well-known since Einstein's derivation of the diffusion equation based on random walk models (Einstein, 1905).

Several more recent studies serve as practical examples showing that Einstein's homogenization approach for the derivation of the diffusion equation based on random walk scenarios applies also to the case of random walk based calcium dynamics, such that the diffusion-reaction equation approach even is justified in the presence of bi-molecular binding interactions (Hake and Lines, 2008; Modchang et al., 2010; Matveev, 2021).

Our mathematical model consists of two concentrations, namely calcium and a calcium buffer. As in general, calbindin is the major buffer of calcium within presynaptic regions, we assume that the buffer substance is calbindin (Nadkarni et al., 2010). Anyhow, as we are dealing with a mathematical model, our choice of calbindin does not exclude other possible buffer types such as calmodulin (Pang et al., 2010), which is the major buffer in postsynaptic zones. Indeed, the mathematical coefficients which describe the binding of calcium to calbindin and calmodulin are not completely different, and we will check our computations for a wide range of reaction rates for binding and unbinding of calcium to the respective buffer. Therefore, our model also covers the possibility that calmodulin would be the major buffer, or that we would have a mixture of different buffer substances. Hence, we created a model where calcium and calbindin (or any other generic buffer) are diffusing inside the 3D computational domain, and calcium and calbindin are interacting *via* a reactive term, i.e., calcium is buffered. Thus, we have to compute the equations


(1) $$\begin{array}{l} \frac{\partial }{\partial t}\left[Ca^{2+}\right]\left(\vec{x},t\right)=D_{Ca}\Delta \left[Ca^{2+}\right]\left(\vec{x},t\right)-R\left(\vec{x},t\right)\;\forall \vec{x}\in V \end{array}$$

(2) $$\begin{array}{l} \frac{\partial }{\partial t}\left[B\right]\left(\vec{x},t\right)=D_{B}\Delta \left[B\right]\left(\vec{x},t\right)-R\left(\vec{x},t\right)\;\forall \vec{x}\in V \end{array}$$

for the concentrations of calcium [*Ca*^2+^] and the free buffer calbindin [*B*] (assuming the constant total amount of buffer [*B*]_*total*_ such that the bounded calcium buffer complex concentration is the difference of total and free calbindin [*B*]_*total*_ − [*B*]) (Neher, 1995; Bazhenov et al., 2004; Müller et al., 2005; Breit and Queiser, 2018), which are computed at each point of the 3D mesh, i.e., at all $\vec{x}\in V$ at each time point *t* of the simulation time.

The kinetic reactions follow the law of mass action (LaMA) (Neher, 1995; Bazhenov et al., 2004; Müller et al., 2005; Breit and Queiser, 2018):


(3) $$\begin{array}{l} R\left(\vec{x},t\right)=k_{on}\left[B\right]\left(\vec{x},t\right)\left[Ca^{2+}\right]\left(\vec{x},t\right)-k_{off}\left({\left[B\right]}_{total}-\left[B\right]\left(\vec{x},t\right)\right) \end{array}$$

*D*_*Ca*_ and *D*_*B*_ are the diffusion constants of calcium and calbindin, respectively. For the exact values of the parameters, we refer to Table 1.

**Table 1:** Parameters of the partial differential equation (PDE) simulations Equations (1), (2).

**Parameter**	**Value**	**Unit**	**References**
*D*_*Ca*_	200	[μm^2^ s^−1^]	Allbritton et al., 1992; Nadkarni et al., 2010
*D*_*B*_	30	[μm^2^ s^−1^]	Luby-Phelps et al., 1995; Nadkarni et al., 2010
*k*_*on*_	0 - 44	[s^−1^ (μM)^−1^]	Nadkarni et al., 2010
*k*_*off*_	0 - 36	[s^−1^]	Nadkarni et al., 2010
${\left[Ca^{2+}\right]}_{V}^{0}$	50	[nM]	Schneggenburger and Neher, 2005
[*B*]_*total*_	40	[μM]	Neher, 1995; Müller et al., 2005
${\left[Ca^{2+}\right]}_{e}$	1.5	[mM]	Egelman and Montague, 1999
*I*_*p*_	8·10^−24^	[mol/s]	Graupner, 2003
*H*_*p*_	60	[nM]	Elwess et al., 1997
*n*_*p*_	2		Graupner, 2003
*I*_*x*_	2.5·10^−21^	[mol/s]	Graupner, 2003
*H*_*x*_	1.8	[μM]	Graupner, 2003
*n*_*x*_	1		Graupner, 2003

*Note that for various parameters, we use different values for the sake of a sensitivity analysis*.

Furthermore, we use the notations


(4) $$\begin{array}{l} {\left[Ca^{2+}\right]}_{i}\equiv \left[Ca^{2+}\right] \end{array}$$

(5) $$\begin{array}{l} {\left[Ca^{2+}\right]}_{e}\equiv \text{const} \end{array}$$

That is, we assume constant extracellular calcium concentration ${\left[Ca^{2+}\right]}_{e}$, “calcium extracellular” (often also called ${\left[Ca^{2+}\right]}_{o}$, for calcium outside) in the synaptic cleft (as the concentration there is much larger than inside the presynaptic bouton), and the internal calcium concentration ${\left[Ca^{2+}\right]}_{i}$ is the concentration we compute by means of our PDE (1), [*Ca*^2+^] combined with the initial and boundary conditions to be explained next:

As the initial condition for the concentrations inside the computational domain, we choose constant values all over the computational domain, which are based on the values given in Table 1. Indeed, using the dissociation coefficient (Matthews and Dietrich, 2015).


(6) $$\begin{array}{l} K_{D}=\frac{k_{off}}{k_{on}}, \end{array}$$

the initial values of free calcium and free buffer are computed as Neher (1995) and Müller et al. (2005).


(7) $$\begin{array}{l} \left[Ca^{2+}\right]\left(\vec{x},t=0\right)={\left[Ca^{2+}\right]}_{V}^{0} \end{array}$$

(8) $$\begin{array}{l} \;\left[B\right]\left(\vec{x},t=0\right)={\left[B\right]}_{V}^{0}=\frac{{\left[B\right]}_{total}K_{D}}{{\left[Ca^{2+}\right]}_{V}^{0}+K_{D}} \end{array}$$

At the sides and the top of the computational domain, i.e., at W, for all surrounding rectangles of the rectangular cuboid except for the ground, we impose Dirichlet boundary conditions aiming the initial values of calcium and the buffer,


(9) $$\begin{array}{l} \begin{array}{c} \left[Ca^{2+}\right]\left(\vec{x},t\right)={\left[Ca^{2+}\right]}_{V}^{0}\\ \left[B\right]\left(\vec{x},t\right)={\left[B\right]}_{V}^{0} \end{array}\}\;\forall \vec{x}\in W \end{array}$$

as we assume the bouton to be sufficiently large so that concerning short-term plasticity, the VGCC caused calcium influx will not have a substantial influx upon the total calcium concentration inside the bouton but just for the calcium microdomains at the AZ.

At the ground of the cuboid, we impose Neumann flux conditions.^1^

Except for the case of channel zone C, we have Neumann zero flux boundary conditions at the bottom B of the covering cuboid. This means that at the boundaries, we have Neumann zero conditions at the bottom surface of the surface/rectangle of the surrounding rectangular cuboid (where the T-bar is grounded) B, also at the surface of the T-bar itself, T.

At the surface where the VGCCs are located, i.e., at the channel zones C (cf. Figures 1B–D), we have Neumann influx of calcium governed by means of N-type VGCCs (Borg-Graham, 1998), PMCA (Graupner et al., 2005) and NCX membrane transporters (Yu and Choi, 1997), and leak currents (Cuttle et al., 1998) caused by the membrane transporters.

Written in a closed form, the Neumann flux boundary conditions read:


(10) $$\begin{array}{l} \vec{n}\cdot D_{Ca}\nabla \left[Ca^{2+}\right]\left(\vec{x},t\right)=\{\begin{array}{cc} I_{M}\left(V,t,{\left[Ca^{2+}\right]}_{i},{\left[Ca^{2+}\right]}_{e}\right) & \forall \vec{x}\in C\\ 0, & \forall \vec{x}\in B\cup T \end{array} \end{array}$$

with the membrane current given per surface unit


(11) $$\begin{array}{l} I_{M}\left(V,t,{\left[Ca^{2+}\right]}_{i},{\left[Ca^{2+}\right]}_{e}\right)=\rho_{VGCC}\cdot I_{VGCC}\left(V,t,{\left[Ca^{2+}\right]}_{i},{\left[Ca^{2+}\right]}_{e}\right)\\ \;+\;\rho_{PMCA}\cdot I_{PMCA}\left(t,{\left[Ca^{2+}\right]}_{i},{\left[Ca^{2+}\right]}_{e}\right)\\ \;+\;\rho_{NCX}\cdot I_{NCX}\left(t,{\left[Ca^{2+}\right]}_{i},{\left[Ca^{2+}\right]}_{e}\right)\\ \;+\;\rho_{leak}\cdot I_{leak}\left(t,{\left[Ca^{2+}\right]}_{i},{\left[Ca^{2+}\right]}_{e}\right) \end{array}$$

The membrane current/flux is the superposition of the voltage-gated calcium (VGCC) influx current/flux $I_{VGCC}\left(V,t,{\left[Ca^{2+}\right]}_{i},{\left[Ca^{2+}\right]}_{e}\right)$ with the Ca^2+^-ATPase pumps (PMCA) current/flux $I_{PMCA}\left(t,{\left[Ca^{2+}\right]}_{i},{\left[Ca^{2+}\right]}_{e}\right)$ and the Na^+^ /Ca^2+^ exchangers (NCX) current/flux $I_{NCX}\left(t,{\left[Ca^{2+}\right]}_{i},{\left[Ca^{2+}\right]}_{e}\right)$ and the membrane leakage current/flux $I_{leak}\left(t,{\left[Ca^{2+}\right]}_{i},{\left[Ca^{2+}\right]}_{e}\right)$. PMCA and NCX current/fluxes are outwards, they pump calcium out of the presynaptic AZ into the synaptic cleft. The leakage current/flux takes care of the current so that in equilibrium state, when there is no action potential, there is no net flux over the membrane.

Note that the membrane streams are multiplied with their corresponding densities per unit area. For example, the density of the VGCC channels ρ_*VGCC*_ is given in [μ*m*^−2^].

To describe the VGCCs of N-type calcium channels, we use the model published in Borg-Graham (1998), for details cf. Section 2.3. PMCA and NCX pumps parameters are described in Section 2.4. We recall that the calcium pumps act and leakage fluxes take place only in the regions where the channels are located, i.e., at the flux surface C.

The buffers, pumps, and leakage are incorporated technically as described by Graupner (2003) and Breit and Queiser (2018).

### 2.3. Voltage Gated Model of Calcium Influx

#### 2.3.1. VGCC Ion Current/Flux Dynamics

The dynamics of ionic fluxes of VGCC in the case of a neuron membrane entering (11) is described in the following way (published in Borg-Graham 1998):


(12) $$\begin{array}{l} I_{I}\left(V,t,{\left[Ca^{2+}\right]}_{i},{\left[Ca^{2+}\right]}_{e}\right)=G\left(V,t\right)F\left(V,\Delta X\right) \end{array}$$

Thus, we have a product of a (or several) gating functions *G* and flux functions (which would be valid in the case that all gates are open) F.

#### 2.3.2. Flux Functions

The current for a single channel, i.e., the flux of calcium can be described as by means of the Goldman-Hodgkin-Katz equation (GHK) (Jack et al., 1983; Hille, 1992):


(13) $$\begin{array}{l} F\left(V,{\left[Ca^{2+}\right]}_{i},{\left[Ca^{2+}\right]}_{e}\right)\\ =p_{x}\frac{z^{2}F^{2}V}{RT}\left[\frac{{\left[Ca^{2+}\right]}_{i}-{\left[Ca^{2+}\right]}_{e}\exp\left(-z\frac{FV}{RT}\right)}{1-\exp\left(-z\frac{FV}{RT}\right)}\right] \end{array}$$

is given in [A] (Ampère), where *p*_*x*_ is the permeability, *T* is the temperature, *V* is the voltage, *R* is the gas constant, and *F* is the Faraday constant. The physical constants used are the Faraday constant *F* = 9.648·10^4^ C mol^−1^, and the gas constant *R* = 8.314 V C K^−1^ mol^−1^. We will set the temperature to *T*= 300 K, corresponding to 27 °C.

#### 2.3.3. Gating Functions

The gating functions may be a product of several functions of type, usually of the form


(14) $$\begin{array}{l} G\left(V,t\right)=m^{a}\left(V,t\right)h^{b}\left(V,t\right)=\overset{j_{max}}{\underset{j=0}{\prod }}x_{j} \end{array}$$

The powers of *m, h*, i.e., *a, b*, depend on the special channel.

The *x*_*j*_ fulfill ordinary differential equations (ODEs) of type (we write generically *x*)


(15) $$\begin{array}{l} \frac{\partial x}{\partial t}=\frac{x_{\infty }\left(V\right)-x}{\tau_{x}\left(V\right)} \end{array}$$

which is a Hodgkin-Huxley-like model, adapted for calcium and other ion types by Borg-Graham (1998). For details of the model, as well as for the parameters used in the case of calcium N-type channels, we refer to Borg-Graham (1998), namely also the parameters given by Borg-Graham (1998), Table 5, page 95.

#### 2.3.4. Channel Numbers, Distribution, and Sensitivity Analysis

We assume that the VGCCs are distributed continuously over the surface C for the two different configurations, clustered and not clustered channel distribution. This indicates that the VGCC density has the same value all over the respective channel zones for the given anatomy. We apply a different channel density for the two geometric cases of channel structure, with and without clustering, but the same number of total channels for all three anatomic scenarios.

In both cases, we assume 6 VGCC calcium channels of N-type (Borg-Graham, 1998) as standard setup. The surface and the densities where the flux is enabled for the two different possible channel distribution cases hence read for the case of 6 VGCCs at the AZ:

- The channel surface C in case clustered channel distribution is assumed to be 0.0026 μm^2^ and the VGCC density ρ_*VGCC*_, hence, is 2297.1 μm^−2^.
- The channel surface C in case of unclustered channel distribution is assumed to be 0.015 μm^2^ and the VGCC density ρ_*VGCC*_, hence, is 395.8 μm^−2^.

Note that in the case of not clustered channels, the overall surface covered by the channels is larger, such that the total number of channels is the same in all cases.

Despite the fact that for our standard parameter setup, we assume 6 VGCCs per AZ for the main results, we also performed a sensitivity analysis using other numbers of VGCCs per AZ: Since the channel number is only an estimation and has not been measured experimentally to our knowledge, we investigate the sensitivity of our results with respect to the channel number. We probe for a wide range of other numbers of VGCCs per (AZ), ranging from only 1 up to 200, as Nadkarni et al. (2010) reports this wide range of possible number of VGCCs to look at the impact of the channel number upon the calcium microdomain structure. Namely, we investigate if a change in the number of the VGCCs has an impact upon the major results of our study.

Nevertheless, we assume that the number of VGCCs at the Drosophila larval NMJ will be much lower than 200 per AZ, and presumably is in the region between 1 up to maximally 10. Our results reported below show results for the variation of the VGCCs in the range of Nadkarni et al. (2010) to ensure the broad validity of our approach nevertheless.

### 2.4. PMCA and NCX Pumps and Leakage

For the PMCA and the NCX current (Graupner, 2003; Meyer-Hermann et al., 2003), we have


(16) $$\begin{array}{l} I_{PMCA}=I_{p}\frac{{\left({\left[Ca^{2+}\right]}_{i}\right)}^{n_{p}}}{H_{p}^{n_{p}}+{\left({\left[Ca^{2+}\right]}_{i}\right)}^{n_{p}}} \end{array}$$

(17) $$\begin{array}{l} I_{NCX}=I_{x}\frac{{\left({\left[Ca^{2+}\right]}_{i}\right)}^{n_{x}}}{H_{x}^{n_{x}}+{\left({\left[Ca^{2+}\right]}_{i}\right)}^{n_{p}}} \end{array}$$

with the values given in Table 1.

Note that following (Graupner, 2003), we use a Hill coefficient of 2 for the PMCA pumps, the Hill coefficient for the NCX pumps is set to 1. Note that when calculating the coefficients *I*_*p*_, *I*_*x*_ based on the values given in Graupner (2003), but defined there as multiplied with *zF*, we have used *z* = 2, as Ca^2+^ has valence two.

For the density of the PMCA pumps ρ_*PCMA*_, we perform the computations for maximum values originating in literature, but also compute for vanishing PMCA pumps to unveil the impact of the pumps upon the basic question of the relation of form and function in the context of this synapse. Hence, we use the minimum value 0 and the maximum value 75, 000μ*m*^−2^, which covers completely the reported values (Chen et al., 2012). For the density of the NCX pumps ρ_*NCX*_, we apply the same technique and use the minimal value 0 and the maximal value 2, 500μ*m*^−2^, which as well covers completely the reported values (Frank et al., 1992).

The leak current density and the leak current were adjusted such that at rest, inner calcium concentration is at equilibrium in the absence of action potentials.

### 2.5. Buffer Reaction Coefficient Sensitivity Analysis

In this study, we considered as well the case with nonzero buffering constant *k*_*on*_ and unbuffering constant *k*_*off*_ based on literature values, and vanishing buffer reactions and vanishing pumps and leakage, to check the complete spectrum of calcium concentrations for all possible buffer and pump scenarios, i.e., to probe the influence of buffers and pumps onto the overall calcium microdomain dynamics at this synapse. The buffer reaction coefficient is taken from literature by Müller et al. (2005), but we also compute with vanishing buffer reactions, i.e., for “inert” buffers. The buffer reaction model itself arises from Neher (1995). The vanishing buffering mechanism, i.e., vanishing reaction, we use as a comparison case.

The major studies, we perform with the case with the buffer reaction parameters from the literature, as this case reflects the “worst-case” scenario for concentrations to develop a difference between the geometries - if more calcium gets bound, the diffusion barrier of the T-bar anatomical structure likely will have less influence on the calcium accumulation at the active zone compared to the case without calcium reduction due to bindings to the buffer.

### 2.6. Parameter Table

The parameters used in this study and their respective literature basis are reported in Table 1. Note that in the case of several parameters, namely those in the context of buffers and pumps, we checked for minimum and maximum values, to study their respective influence.

### 2.7. Form of Repetitive Action Potential

We impose an action potential at the synaptic membrane “felt” by the calcium channels. The action potential has a typical form and is displayed by (Supplementary Figure S1, cf. Supplementary Material) for the case of 40 Hz. For the other cases of stimulation frequencies, the stimulus frequency changes, but not the entire shape. Each single action potential contributes to the calcium microdomain concentration.

### 2.8. Spatial Regions for Quantitative Evaluations

**Figure 3:**
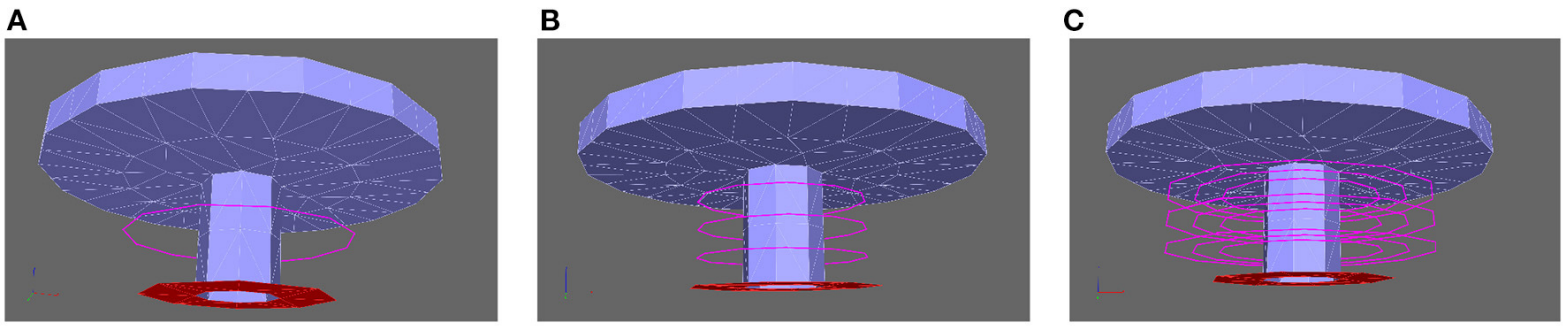
Aim of this figure: Display the regions where the calcium concentrations will be evaluated which arise within our simulations. The regions of evaluations are circles around the T-bar “socket”. The circles are placed such that they cover the region below the T-bar “roof” in a rather dense way, as well in height computed from the membrane base, and also in distance from the T-bar “socket”. In the Results, Section 3, we evaluate the averaged concentrations of each circle, as evaluations at only single points might not be so favorable, even though we also check that our results are independent of the computational grid. Constructing circles around the T-bar socket to measure calcium at equidistant radii and heights from the T-bar socket. Note the thin magenta colored lines around the table leg, which mark the evaluation lines. Circles constructed based upon (18). **(A)**: One circle, **(B)**: three circles for fixed radius, but different height, **(C)**: various circles for different heights and different radii.

Among the simulations, we compared the arising calcium concentrations over time, and depending on the distance from the center of the AZs, we created circles around the T-bar socket with different heights, to evaluate in later steps the calcium concentrations for specific radii (distance from the T-bar socket) and heights (measured from the ground of the AZ, i.e., the membrane). Figure 3 depicts some of the circles, where we evaluate the concentration of calcium within our simulations. Indeed, the different circles are constructed at the following zones:


(18) $$\begin{array}{l} \vec{K}\left(n_{h},n_{r},n_{\phi }\right)={\vec{b}}_{T}+\left(\begin{array}{c} n_{r}\cdot \Delta_{r}\cdot \cos\left(2\pi \frac{n_{\phi }}{n_{\phi }^{max}}\right)\\ n_{r}\cdot \Delta_{r}\cdot \sin\left(2\pi \frac{n_{\phi }}{n_{\phi }^{max}}\right)\\ n_{h}\cdot \Delta_{h} \end{array}\right) \end{array}$$

where ${\vec{b}}_{T}$ is the basis of the T-bar leg in the membrane, i.e., the midpoint at the membrane, where the T-bar socket is located, and we use


(19) $$\begin{array}{l} n_{h}=1,2,3,\ldots ,n_{h}^{max} \end{array}$$

(20) $$\begin{array}{l} n_{r}=1,2,3,\ldots ,n_{r}^{max} \end{array}$$

(21) $$\begin{array}{l} n_{\phi }=0,1,2,3,\ldots ,n_{\phi }^{max}-1 \end{array}$$

where we choose Δ_*h*_ and Δ_*r*_ such that we have a raster covering of the space below the T-bar zone, but $n_{\phi }^{max}$ such that the arising circles at different heights and radii are quite fine resolved. Note that Δ_*h*_ and Δ_*r*_ are simply spatial lengths measures, as they mark the differences between different raster of height and radius. This means that we are covering the zone around the T-bar “socket” with circles of different heights above the membrane, and with different radii. The midpoint of the radii is the midpoint of the T-bar “socket.” Note that due to the fact that depending on the choice of Δ_*r*_, the T-bar socket might cover a part of the evaluation points, of course, we have to choose the minimum value of *n*_*r*_ such that the evaluation radius is located outside the socket, indicating that the minimal value of *n*_*r*_ might be bigger than one, but necessarily a natural number. In more detail, the equation describing the circle's bundle $\vec{K}\left(n_{h},n_{r},n_{\phi }\right)$ is the combination of the base vector ${\vec{b}}_{T}$ which is the localization where the T-bar grows at the membrane. The second part,


$$\begin{array}{l} \left(\begin{array}{c} n_{r}\cdot \Delta_{r}\cdot \cos\left(2\pi \frac{n_{\phi }}{n_{\phi }^{max}}\right)\\ n_{r}\cdot \Delta_{r}\cdot \sin\left(2\pi \frac{n_{\phi }}{n_{\phi }^{max}}\right)\\ n_{h}\cdot \Delta_{h} \end{array}\right) \end{array}$$

is the vector to the single circles, and it is given by means of cylinder coordinates. This means that in the first and second directions, we have spherical coordinates in two dimensions, whereas in the third coordinate, we have a simple value. The spherical coordinates describe a circle in two dimensions with a given radius around the center, which is given by the third component vector. The different values of *n*_*h*_, *n*_*r*_, and *n*_ϕ_ allow for a discretized version of the circles in space, i.e., described by single but close points. The values for *n*_*r*_ and *n*_*h*_ refer to the raster pattern of pairwise distinct circles, whereas *n*_ϕ_ refers to the discrete points of each circle (as we need to discretize the circles). The values for *n*_*r*_ and *n*_*h*_ are comparably small numbers, while the values for *n*_ϕ_ may reach higher values, depending on the resolution of the single circle, which is given by $n_{\phi }^{max}$. For more details concerning cylinder coordinates, we refer to the literature cf. e.g. Spiegel (1976).

### 2.9. Technical Details

#### 2.9.1. Numerical Methods to Solve the PDEs

The PDEs are discretized by means of a vertex-centered finite volume scheme for unstructured grids and solved by means of a Newton solver. The arising linear equations are solved with the aid of a BiCGSTAB solver preconditioned with a geometric multigrid GMG.

Our simulations are performed with the simulation framework UG4 (Heppner et al., 2013; Vogel et al., 2013), which has its origin in the field of entire applied mathematics, and has already proven high efficacy for various fields in applied mathematics, geosciences, computational virology, and computational neurosciences (cf. e.g., Knodel et al., 2014, 2017, 2018, 2019). This enables us to show the deep foundation of our *in silico* results in a profound manner, also in the sense of standards applied outside life sciences.

#### 2.9.2. Finite Volumes and Evaluation Points

Note that at the first glance, one could get the impression that in Equation (13), the value of intracellular calcium $\left({\left[Ca^{2+}\right]}_{i}\right)$ in the GHK equation would be evaluated within a finite volume immediately next to the calcium channel mouth, and this feature of the model might make the results highly dependent of the chosen grid resolution, such that at the end, the solution strongly would depend on the element under consideration.

However, indeed, this does not hold true for that type of the vertex centered finite volume methods we use, as we apply a weak formulation-based approach (Bey, 1998; Ern and Guermond, 2004; Vogel et al., 2010).

Within the weak solution-based vertex-centered finite volume approach, the solutions are not simply computed element by element, but moreover in a continuous manner. In particular, the starting point of weak methods based on finite volume or finite element approaches is *not* the discrete space of the elements of the grid, but moreover the continuous equation. The discrete basis of solutions is established by means of the solution space which is realized by means of Sobolev spaces. Therefore, the solution is computed in a continuous manner all over the computational domain. For details of the theory, we refer to the literature such as Bey (1998), Ern and Guermond (2004), and Vogel et al. (2010).

To ensure numerical robustness, numerical grid convergence tests can be applied, and we perform this procedure to ensure numerical grid convergence of our results.

#### 2.9.3. DoF Number

The number of degrees of freedom (DoFs) for the geometries and the corresponding refinement level of the 3D tetrahedral volume mesh are displayed in Table 2. Note that the geometry itself is the same for the case of no T-bar but either clustered or not clustered channel zone - the difference between the two cases refers to the region where influx is permitted and the corresponding channel density.

**Table 2:** Degree of freedom (DoF) number for the different geometric configuration volume meshes, for the different levels of grid refinement.

**Level**	**T-bar**	**no T-bar**
0	690	542
1	4,778	3,842
2	35,018	28,890
3	266,802	223,954
4	2,079,970	1,763,362

### 2.10. Technical Framework in the Context of *in silico* Approaches

Several years ago, there were attempts to model the interplay of calcium and vesicle dynamics based on 3D PDEs, which were solved by simplifying the equations for enabling analytic solutions by Fogelson and Zucker (1985) and Parnas et al. (1989), as techniques from mathematical numerics were not standard in computational neuroscience. This lack is vanishing more and more in recent years, PDEs and numerical solution techniques developed in the field of applied mathematics are getting more and more spread in the computational neuroscience community. Nevertheless, important results still have to be based on simple ODE modeling techniques like (Fink et al., 2014). The “standard” compartmentalization method is implemented in various packages from the community like NEURON (Carnevale and Hines, 2006). They were used efficiently by many groups like e.g., Segev and connected groups (Yaron-Jakoubovitch et al., 2008; Sarid et al., 2013; Eyal et al., 2014). Compartmentalization methods are also used independently of program packages (e.g., De Young and Keizer, 1992; Bertram et al., 1996; Keizer and Levine, 1996; Berggard et al., 2002; Cannon et al., 2010). The compartmental method and simplified PDE methods (like 2D Finite Differences (FD) and symmetric solutions in space) were used e.g., by Neher, Sakmann and respective connected groups for the description of calcium dynamics effects (Naraghi and Neher, 1997; Naraghi et al., 1998; Koester and Sakmann, 2000; Meinrenken et al., 2002, 2003; Taschenberger et al., 2005; Hosoi et al., 2007; Wölfel et al., 2007). Also, other groups followed the kinetic ODE compartment ansatz like (Jaffe et al., 1994; Smith et al., 1996; Borg-Graham, 1998; Jones, 1998; Erler et al., 2004; Graupner et al., 2005; Pradhan et al., 2010, 2011). In many cases, the 3D PDEs are adapted to simplified cases which afterward allow for solutions of the equations (c.f. e.g., van Hemmen Lüling et al., 2010 or Makram/Helmchen Markram et al., 1998). De Schutter et al. calculated various processes (Bormann et al., 2001; Hepburn et al., 2013) with their Monte Carlo based STEPS package (Hepburn et al., 2012; Chen and De Schutter, 2014) which is exclusively written for calcium processes. De Schutter further also used simplified compartment/ODE methods (Anwar et al., 2012) and reduced one dimensional spatial models (Santamaria et al., 2011). The packages NeuroRD (Oliveira et al., 2010) and Cell motility (Martens et al., 2006) also are based on stochastic Monte Carlo simulations of random walks. Also, other groups are using Monte Carlo methods for PDE solutions like (Kennedy et al., 1999; Kerr et al., 2008; Andrews et al., 2010; Nadkarni et al., 2010) for vesicle and/or calcium dynamics evaluation. The program package MCELL (Stiles and Bartol, 2001) is based on Monte Carlo methods, in principle thought for various scenarios. More recently, the FD based 3D PDE package CalC (Matveev, 2009; Matveev et al., 2009) for the sake of calcium calculations is used widely, e.g., by Wolf and Moser (Frank et al., 2009; Wong et al., 2014), caused by the need of calcium 3D PDE solutions. The package VCELL (Virtual Cell) incorporates not only compartment simplifications of spatially resolved processes but also stochastic random walk simulations and structured grid based solvers of PDEs (Cowan et al., 2012; Schaff et al., 2012).

All these examples, algorithm developments and program codes are caused by the need to understand the relation of form and function of e.g., synaptic processes at a cellular level. Of course, the list of articles and programs in the field is not intended to be complete, as here, we do not write a review but just want to give typical examples of the field.

Of course, we do not claim that our simulation framework is superior to the others used in the field. Nevertheless, we believe our framework helps to consider additional research topics in a new light and complements the standard methods with stimulating techniques.

## 3. Results

### 3.1. Qualitative Evaluation of the Simulations

In order to facilitate qualitative understanding of the mathematically quite complex model scenario described in the Materials and Model Section 2 also for experimental scientists which are not familiar with mathematically complex formulae, but with complex experimental setups, we repeat in brief the major aspects of our simulations before evaluating in detail the simulation results.

#### 3.1.1. Simulation Revisited in Brief

We applied repetitive action potential stimulation to the membrane of the AZ. This action potential stimulation was realized technically by imposing a given time dependent voltage, which was applied to the calcium N-type channels located at the membrane of the AZ. We simulated the influx of calcium into the active zone by means of VGCC calcium N-type channels and the intracellular calcium microdomain concentration dynamics by means of a diffusion-reaction model. We considered the case of three different geometrical scenarios (note that in this context, the geometrical and anatomical scenarios can be considered as synonyms):

- Active zone with T-bar and calcium channels clustered around the T-bar socket, directly at the presynaptic AZ membrane.
- Active zone without T-bar, but still clustered calcium channels around an “imaginative” T-bar socket.
- Active zone without a T-bar, and the calcium channel number is the same as in the two other scenarios, but the channels are distributed over a bigger surface, such that this example realizes the case of not clustered calcium channels.

#### 3.1.2. Qualitative Evaluation - 3D *in silico* Microscope

**Figure 4:**
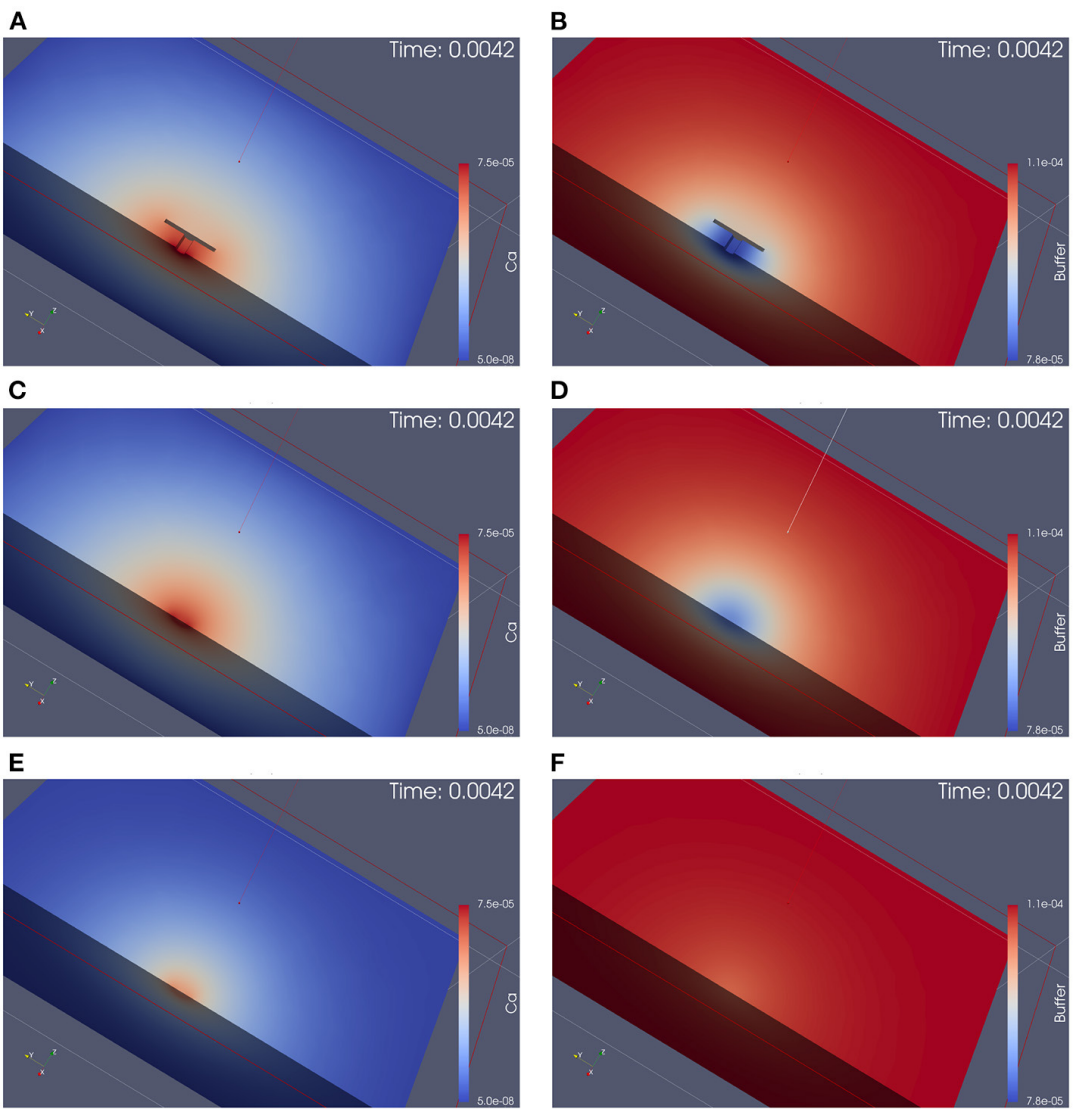
Simulation of calcium and buffer concentration under repetitive action potential stimulation for the three different anatomical cases. Screenshots are taken at the first calcium microdomain peak. Full simulation movies are available as Supplemental Material. The simulation movies show the computational domains disclosed by means of a cut plane. Left: calcium concentration, Right: buffer concentration. From top to down: With T-bar and clustered channels, without T-bar but channels still clustered, without T-bar and no channel clustering. **(A)**: calcium with T-bar, **(B)** buffer with T-bar, **(C)** calcium no T-bar channels clustered, **(D)** buffer no T-bar channels clustered, **(E)** calcium no T-bar channels not clustered, and **(F)** buffer no T-bar channels not clustered. At each action potential, calcium enters through the VGCC calcium channels, diffuses into the presynaptic zone. Due to the limited diffusion speed, calcium accumulates close to the AZ, before it reduces again when the calcium influx reduces again, as the voltage at the membrane drops down. Calcium reduction in major part is due to the diffusion process that causes the calcium to diffuse away. In minor part, calcium buffering and calcium pumps which pump calcium out of the presynaptic bouton again also reduce calcium amount. Before when the next action potential arrives, the calcium level arrives again at initial values (also buffer concentration, which also moves by means of diffusion). At the next action potential, the same process starts again. As the screenshots are taken at peak time, we see easily that in the case of the presence of the T-bar, the concentration at peak time is higher than in the case without T-bar, but still clustered channels, and we observe further that in case without T-bar and without clustered channels, calcium concentration is much smaller. Obviously, the T-bar obstacle causes calcium accumulation below the T-bar, and channel clustering also has a positive influence upon calcium microdomain accumulation.

**Figure 5:**
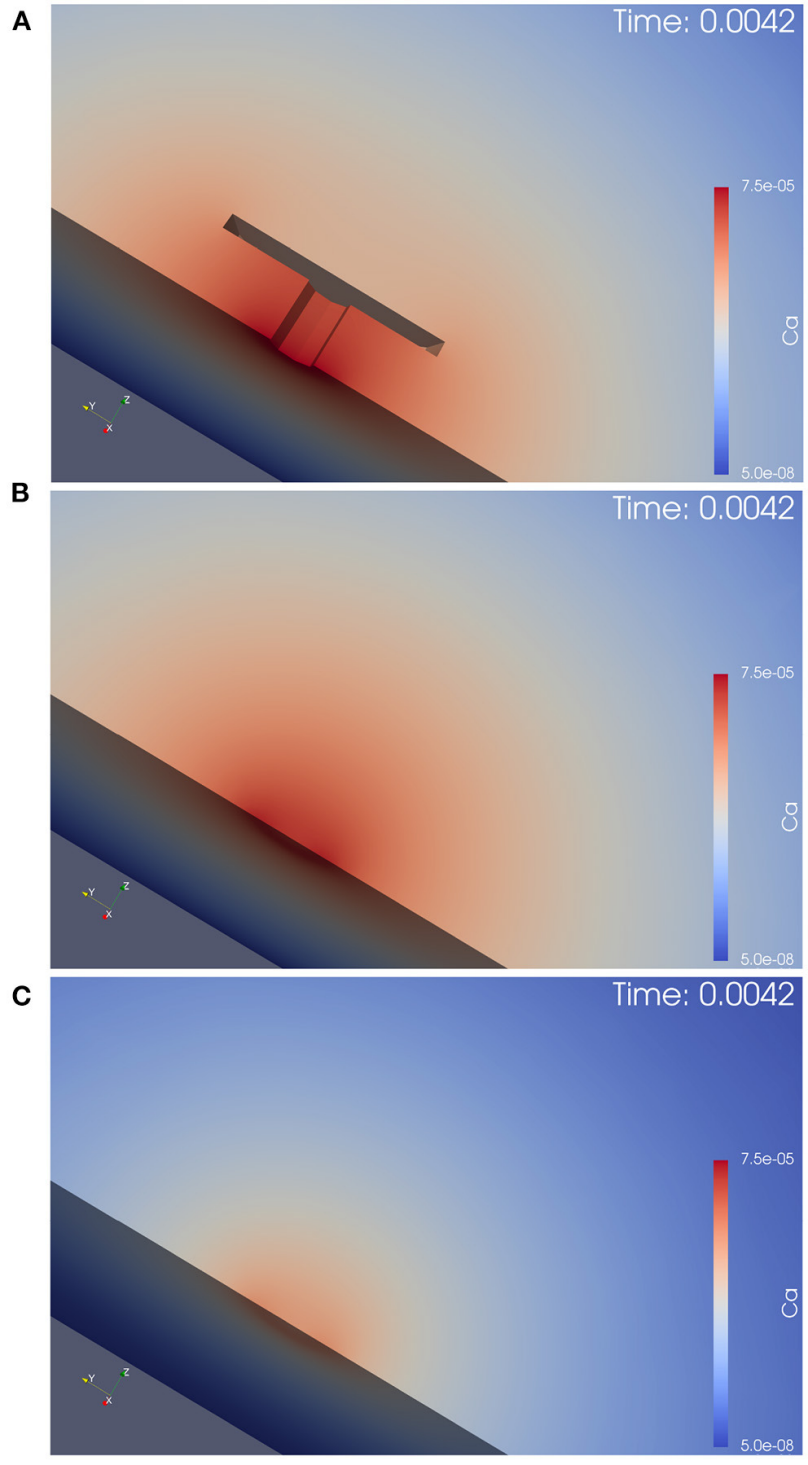
This figure displays the simulation movie screenshot at peak of calcium influx in a zoomed version of the calcium concentration for the three different anatomic scenarios, i.e., it is a magnified version of Figure 4 with the focus on the AZ center, and shows only the calcium, but not the buffer concentrations. We see the computational domain disclosed by a cut plane, and the AZ is in the center. In **(A)**, we see the case of the T-bar with clustered channels, in **(B)**, we see the case without T-bar, but clustered channels. In **(C)**, we see the case without T-bar and without clustered channels. The explanations given in the caption of Figure 4 can be observed here in more detail: The T-bar enhances calcium concentration substantially, while the channel clustering has an additional influence. The lack of each one of these anatomic features reduces the calcium microdomain concentration. Hence, the T-bar and the channel clustering each have a substantial impact upon the calcium microdomain shape.

Figure 4 displays screenshots of the simulations of calcium and the buffer which we performed (the simulation movies are attached as Supplemental Material). Figure 5 displays a zoomed-in version of the calcium concentration simulations of Figure 4 (buffer not displayed).

The 3D simulation movies which we supply can be viewed as some sort of “*in silico* microscope” observation technique, such that the observer can imagine looking through a microscope and observing the spatial and temporal resolved calcium concentration dynamics, where red color means high concentration, blue color small concentration. The scene, which we consider is based upon the complete 3D computational domain opened by means of a cut plane in the middle of the computational domain, such that we can observe the *in silico* experiment in the middle of the zone of interest.

Once an action potential arrives, in all three scenarios, calcium enters the presynaptic bouton close to the AZ. Inside the presynaptic bouton, the calcium concentration moves by means of diffusion and reacts with the buffer. (Note that we also display the free buffer concentration, but in our textual description, we focus upon calcium concentration).

In all three anatomic cases, at each action potential, a calcium microdomain appears close to the channels in the presynaptic boutons.

In all three cases, once when calcium enters, free buffer binds to calcium but has only a limited effect upon the microdomain shape. The free buffer amount decreases once calcium enters and the shape of the buffer concentration mirrors the calcium concentration in an inverse way, as the free buffer decreases when calcium increases.

As calcium influx only appears at the peaks of the action potentials, after when the voltage decreases again, the calcium microdomain disappears quite fast in all three anatomical cases. This means that the calcium in the major part diffuses away, whereas a small part of the calcium gets pumped out again.

As the boutons are considered to be quite big, also the buffer is replenished rather fast by means of diffusion from the other regions of the presynaptic bouton.

So before when the next action potential arrives, the presynaptic calcium and buffer concentrations are already again at effectively the same level as at the very beginning, before when stimulation started.

When the next action potential arrives, the calcium microdomain effect happens again as in the former case.

When comparing the three geometric scenarios using the same scale for the simulations, we see that in the case of the presence of the T-bar, more calcium accumulates close to the channels than in the two cases without T-bar. This is obviously due to the T-bar “table desk” obstacle, which slows down the spread of calcium inside the presynaptic bouton. It is obvious that the T-bar has a major effect on the calcium microdomain peak concentration. Furthermore, comparing the two cases without a T-bar, but with and without clustered channels, we observe that the channel clustering still causes a higher calcium microdomain peak concentration compared to the case when the channels are more widely spread. Also, the channel clustering itself still has a substantial impact.

In conclusion, the combination of T-bar and channeled clusters causes a much higher calcium microdomain concentration compared to the case without T-bar, and even more to the case where also no clustering applies.

This first qualitative evaluation of our simulations already shows a substantial influence of the T-bar structure and even of the clustering structure upon the shape and concentration intensity of the calcium microdomain at each action potential.

This observation is possible already based upon simple evaluations by the view, but it remains to quantify this comparison by means of quantitative measures, which we display in the next sections.

### 3.2. Quantitative Evaluations

#### 3.2.1. Calcium Concentration Profile - Temporal Evaluation at a Fixed Location

In order to quantify the differences in the calcium microdomain concentrations, we perform quantitative evaluations of the computed concentrations for the three given anatomical cases.

**Figure 6:**
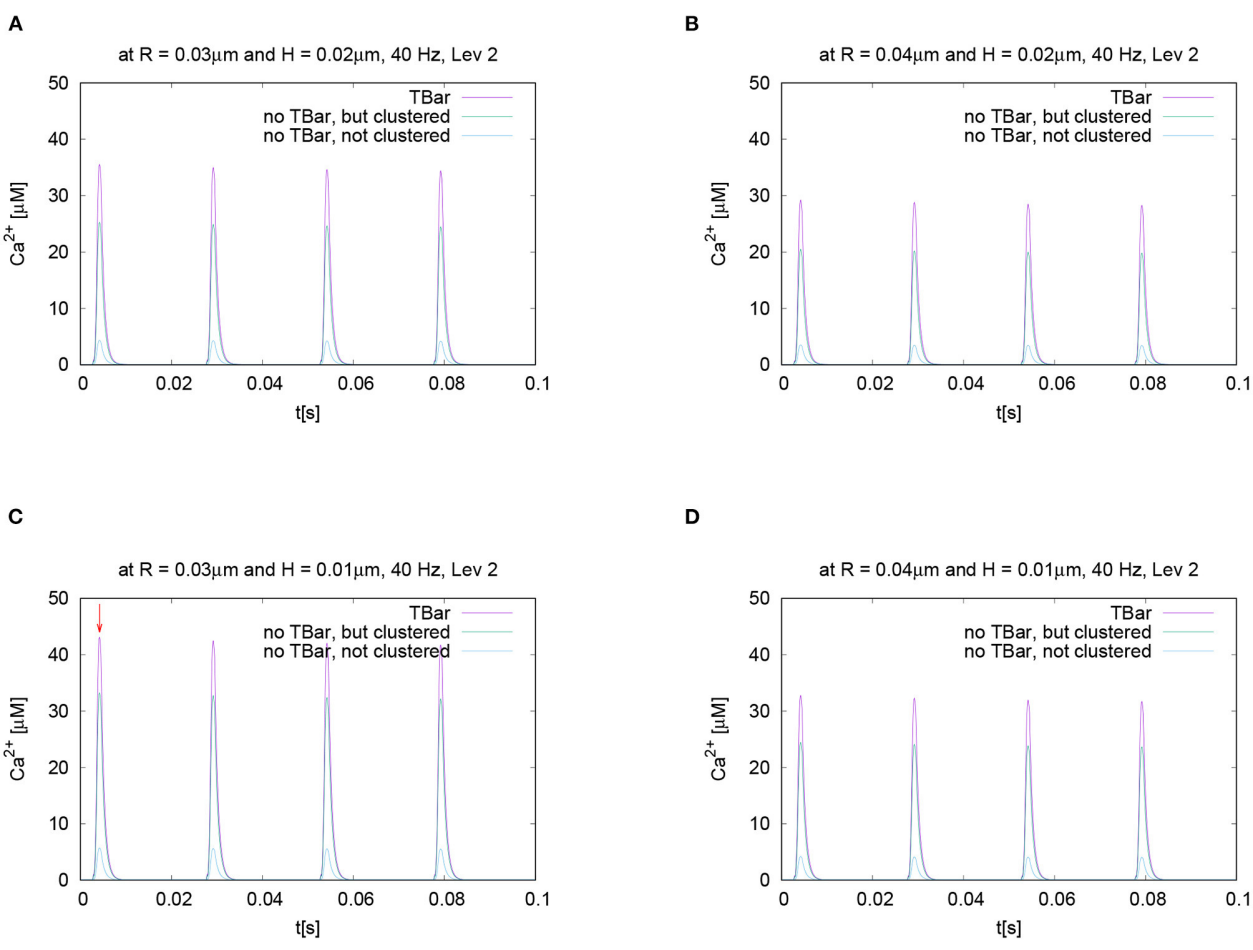
This figure displays quantitative evaluations of the temporal dynamics of calcium concentrations at fixed spatial locations. We display the concentration value of calcium for a given height averaged over a circle with a given radius. The height and the radius are based upon the circles as described in Section 2 and displayed in Figure 3. This means we evaluate the averaged calcium values for a fixed circle. The four sub graphics shown represent the values evaluated at four different circles with noted values of radius and height. Ordering of graphs inspired by their radius and height value i.e., bigger height upper, bigger radius on the right. The radius of calcium concentration evaluation around the (imaginative in the case without T-bar) T-bar socket center at **(C)** radius *R* = 0.03μm and height *H* = 0.01μm, **(A)** *R* = 0.03μm and *H* = 0.02μm, **(D)** *R* = 0.04μm and *H* = 0.01μm, and **(B)** *R* = 0.04μm and *H* = 0.02μm. We see that in all cases, at peak time, the concentration in the case of the T-bar is substantially elevated compared to the case without T-bar, but still clustered channels. Additionally, we see that in all cases, the concentration is the smallest in the case when the channels are not clustered and no T-bar is present. Furthermore, we see that with increasing height, i.e., distance from the membrane of the AZ, the concentrations decrease in all configurations. Also, we see that with increasing radius, the concentration profiles decrease as well.

If we evaluate the temporal evolution of the calcium concentrations at fixed places close to the T-bar (or the corresponding locations without T-bar), i.e., close to the membrane of the AZ, but below the (virtual) T-bar “socket,” averaged for a circle with fixed radius and fixed height, we get profiles over time such as displayed in Figure 6. We recall that the circles of evaluation have their center around the T-bar socket (imaginative in case of no T-bar) for different heights and that each curve refers to the averaged value of the concentration of one of the circles displayed in Figure 3 where we see several examples of circles around the T-bar socket, and we recall the points of evaluation for this circles given in Equation (18).

The evaluation displayed in Figure 6 shows the calcium concentration under action potential stimulation evaluated over time at a given and fixed spatial point below the (virtual) T-bar. We see a substantial difference between the three cases when we consider the peak concentrations which appear at each action potential: the highest calcium concentration is reached in the case when the T-bar is present, followed by the case when there is no T-bar, but channels still clustered. The smallest concentration appears in the case when the channels are not clustered, and there is no T-bar. After when the action potential is finished, the calcium concentration at the given point drops down to the basic value again and rises up in a compatible manner when the next action potential appears.

We see clearly that the case with T-bar has a substantially higher peak than the case without T-bar. In particular, the case without a T-bar and no clustering of channels have a much smaller peak.

As we intend to study the behavior for different radii and heights, Figure 6 displays the time course for different selected values of radii and heights variations for each fixed radius and height value. Based upon the given temporal evaluations at different spatial locations, it seems that the differences between the case of the presence of the T-bar and the absence of the T-bar given that the channels are still clustered depend on the distance to the membrane: The farther away from the membrane we measure, the higher the concentration values seem to differ at the action potential peak. In the case of not clustered channels, the influence seems to be of a minor order.

Nevertheless, it is obvious that this strategy of evaluation over time is not sufficient, and more elaborated calcium profile evaluations have to be considered to understand the spatial profile of calcium dynamics.

#### 3.2.2. Calcium Concentration Profile – Spatial Evaluation at a Fixed (Peak) Time

To investigate the calcium microdomain concentration profile more in detail, we evaluated the concentrations for a fixed time point, at the peak value, and display the variation of the calcium concentration for a fixed height but varying radius, i.e., distance from the (imaginative in case of no T-bar) T-bar socket center.

If not noted otherwise, the evaluations are performed at the first peak marked in Figure 6C. In Section 3.2.3, we consider the comparison for later peaks in detail.

**Figure 7:**
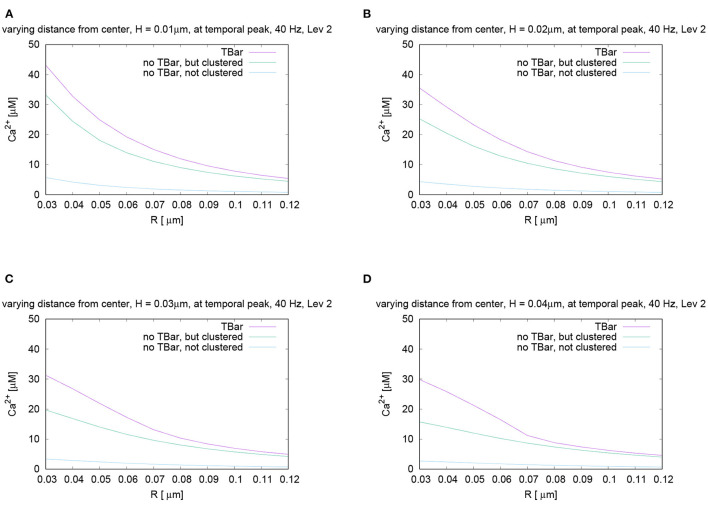
Spatial profiles of calcium concentrations at a fixed time for the three different anatomic cases. The evaluations of the concentrations are performed at the first peak which is marked with a red arrow in Figure 6C, i.e., for *t* = 0.0042s. We evaluate the concentrations for various fixed heights *H* and vary the radius *R*, and display the averaged concentrations over the circles (compare Figure 3) around the (virtual) T-bar socket. Height locations: **(A)** *H* = 0.01μm, **(B)** *H* = 0.02μm, **(C)** *H* = 0.03μm, and **(D)** *H* = 0.04μm. This means that for a given height above the membrane respectively the “root” of the T-bar “socket” (virtual in the case when T-bar is missing), we “walk” from inside to outside for fixed height, and we “jump” from circle to circle. Obviously, in all cases, the concentration is the highest when the T-bar is present, and next, it follows the case when the channels are clustered, but the T-bar is absent. The case of not clustered channels combined with no T-bar shows strongly reduced concentrations in all cases. We observe that in all cases, the concentrations drop down the farther away we go from the center. We see a major impact of the T-bar, namely for small radius and increasing height, i.e., distance from the membrane. If we compare the case of clustered channels with and without T-bar, we observe that as long as we are close to the membrane, for small height, the values differ only by about 25%. However, the more the height grows, the bigger the difference, namely close to the T-bar “socket”. Below the (virtual) T-bar “roof”, the presence of the T-bar causes the concentration to be about a factor two bigger than when the T-bar is missing. This observation indicates that below the T-bar “roof”, vesicles “sense” a much higher calcium concentration, compared to the case without T-bar, even in the case when the channels are clustered. In the case of not clustered channels (without T-bar), the concentrations are reduced even about a factor of 10 compared to the T-bar case. Therefore, this result is a strong hint that the T-bar has quantitatively a strong impact upon the calcium concentration at the AZ.

Figure 7 displays the variation of the calcium concentration for varying radius and heights (i.e., distance from the membrane, higher values more close to the T-bar “roof”).

In the case of clustered channels, with and without T-bar, for each given height, when varying the radius, we observe that the highest value is reached close to the (virtual) socket, whereas we see a drop-down of the concentration when going farther away from the socket. When comparing the clustered channel cases with and without T-bar, we see that close to the membrane, for small height, the values in the case with T-bar are about 25% higher than in the case without T-bar, when we are close to the T-bar socket. The farther we move away from the membrane and closer to the (virtual in case of no T-bar) T-bar “roof,” which means that the height of the evaluation circle becomes larger, the greater the difference gets. Below the T-bar “roof,” the concentration in the case of T-bar anatomy is two times bigger compared to the case where no T-bar is present. When going to higher radii, the differences decrease, as the “blocking” effect of the “roof” has fewer influences, as the sites are open also in the case with T-bar, where the calcium can diffuse away more easily. The clustered channels cause that close to the center, the concentrations are the highest, even without T-bar.

At all spatial locations, the not clustered channel case without T-bar shows concentrations strongly below the case of clustered channels, with or without T-bar. The differences are in the size of magnitude of a factor of about 10, despite the fact that we consider the same number of channels.

The difference in calcium concentration is impressive for the three cases. The variation of the height reveals that the farther away we are from the membrane, i.e., from the channels, the more important becomes the influence of the T-bar “roof.” We see a strong influence on the possibility for calcium to diffuse in the case of the T-bar obstacle present, in the sense that the T-bar indeed acts as a diffusion obstacle.

There are two major sources of different local calcium concentrations: Close to the membrane, the major difference arises from the channel location structure (clustered - not clustered), but the T-bar has an additional non-negligible influence on the peak concentration. Far away from the membrane, close to, i.e., below the T-bar roof (virtual “T-bar” roof in case of no T-bar), the impact of the T-bar is even more important than close to the membrane: Even in the case of clustered channels, there is a strong difference of local calcium concentration between the case with and without T-bar.

#### 3.2.3. Variation of Frequency and Comparison at Later Peaks

**Figure 8:**
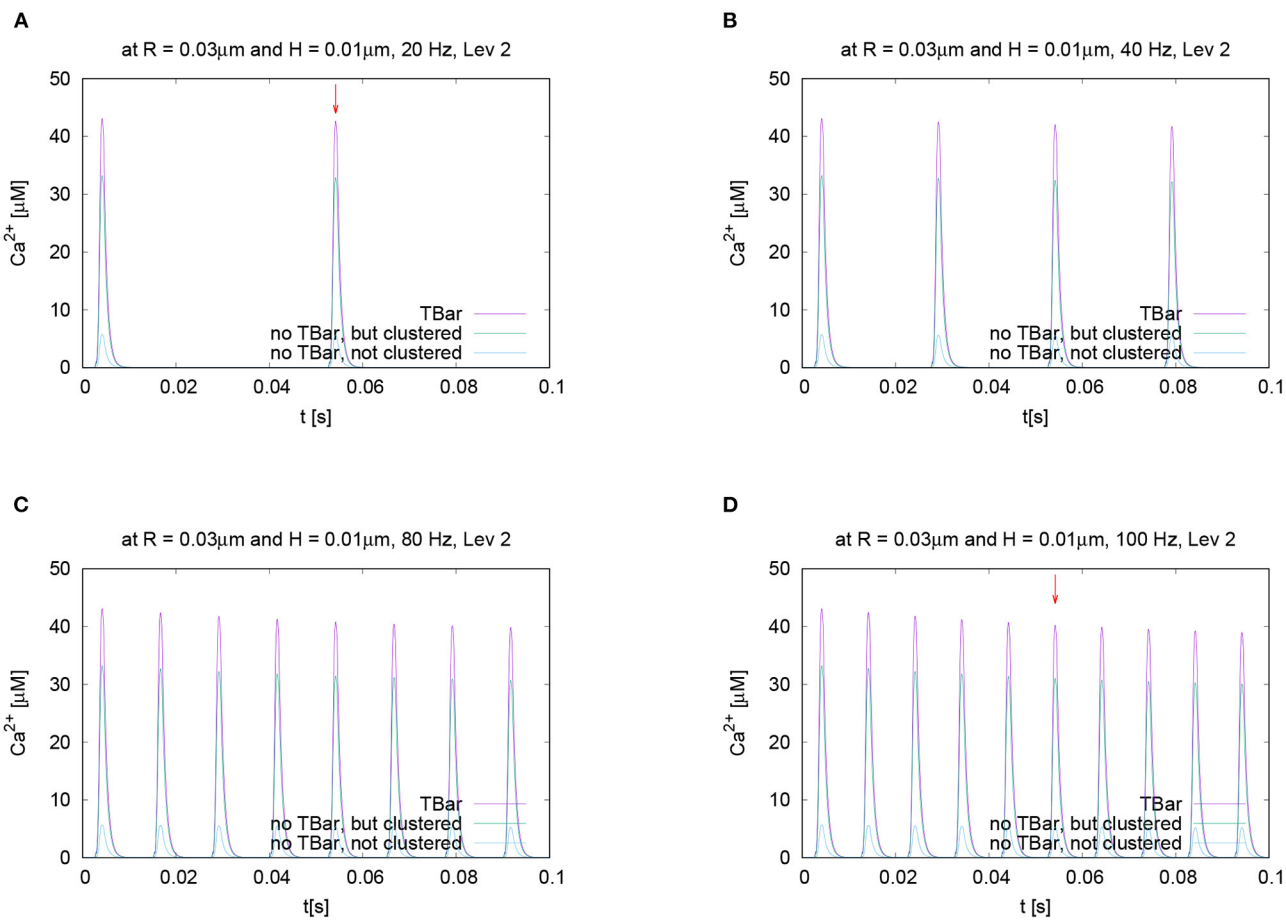
Temporal evolution of the concentration overtime at a fixed spatial location. Fixed height and fixed radius averaged values for different frequencies concentration evaluation around the (imaginative in the case without T-bar) T-bar socket center at radius *R* = 0.03μm and height *H* = 0.01μm. Variation of frequency, comparing high and low frequencies. We observe a very similar behavior at the peaks concerning the relation between the three geometric configurations. Peaks of **(A)** 20 Hz, **(B)** 40 Hz (known case), **(C)** 80 Hz, and **(D)** 100 Hz. stimulation. This figure demonstrates that the variation of frequency has no substantial influence upon the relation of calcium peak concentrations when comparing the three different geometric scenarios. The graphs show that not only at the first peak, but also at later peaks, the T-bar case shows the highest concentration, followed by the case without T-bar, but clustered channels, and that the case without T-bar and with not clustered channels has much less concentration.

In order to test if the frequency of action potential stimulation has an influence upon the relation of the calcium microdomain sizes at the peaks of calcium influx, we varied the frequency of the action potential stimulation. Our question was if maybe at different frequencies, after e.g., several action potentials, the relation of the peak sizes changes for the three different anatomic cases which we consider. To investigate this question, we performed the action potential stimulations with several realistic frequencies: 20 Hz, 40 Hz (i.e., the case already considered before), 80 Hz, and 100 Hz. Figure 8 displays the evaluations of the concentrations evaluating the temporal profile for the (averaged over the circle) value for a given fixed circle around the T-bar “socket” for a given height and radius. We see that all over the simulations, at the action potential peaks, the relations between the three different cases remain effectively the same. We see that the case of the T-bar with clustered channels yields substantially higher peak concentrations of calcium compared to the case of no T-bar, but still channeled clusters. Additionally, we see that the case of no T-bar combined with channels, which are not clustered shows even much smaller peak sizes.

Note that we do not observe calcium accumulation over several action potentials, as the local calcium concentration at the AZ is cleared before the next action potential arrives, also for higher frequencies.

For further quantitative comparisons, we repeated the evaluation method already applied above: We evaluated the concentration profile for a given height, varying the radius at a fixed time point. To perform the comparisons, we evaluated the different curves at the same time point for two different representative frequencies, namely 20 Hz and 100 Hz.

**Figure 9:**
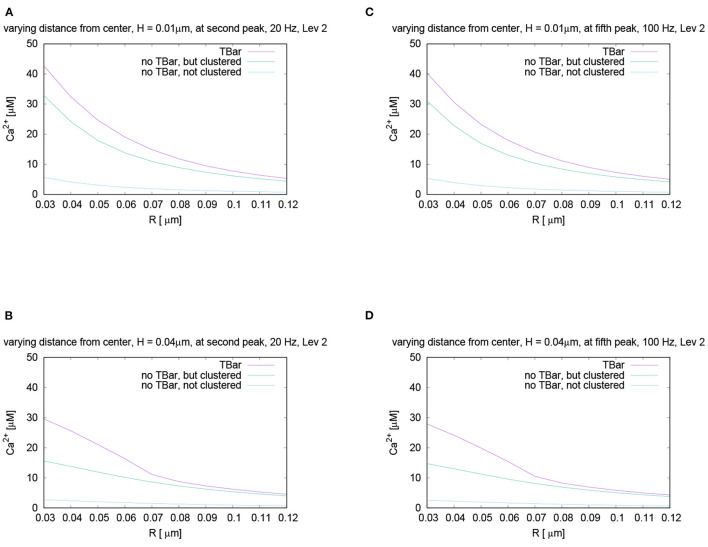
Comparison for the calcium concentration under variation of radius and height at the fixed time point for different stimulation frequencies, i.e., evaluation of the spatial profile of calcium concentrations at peak time after several action potentials, for the same time point. Note the red arrows in Figures 8A,C mark the peaks for which we perform the comparisons. Comparison of the shapes of calcium concentration for the second peak of 20 Hz stimulation **(A,B)**, and the fifth peak of 100 Hz stimulation, which temporally coincides with the second peak of 20 Hz **(C,D)**. We display the profiles for a height close to the membrane, and another one close to the T-bar “roof”. The behavior is practically the same as for the first peak in the 40 Hz stimulation shown in Figure 7. Also here, close to the (virtual) T-bar socket and close to the membrane, the T-bar anatomy shows about 25% more calcium than the case without T-bar but clustered channels. Close to the (virtual) T-bar “roof”, the T-bar case shows about two times more calcium concentration compared to the case without T-bar but clustered channels. As well, the case without T-bar and without clustered channels has much less calcium compared to the cases with clustered channels, i.e., the case of T-bar (with clustered channels always in our model), and the case without T-bar, but clustered channels.

In Figure 8, we mark these time points of evaluation, which correspond to the second peak of the 20 Hz stimulation, and the fifth peak of the 100 Hz stimulation, which coincides temporally. At these peaks, we compare the shapes with varying radii and heights, (cf. Figure 9).

We see that the curves show the same behavior as we have seen before in the case of 40 Hz, (we recall Figure 7): Also for 20 Hz as for 100 Hz, the concentration is the highest for the case with T-bar, then it follows the case without T-bar, but channels clustered. The case without T-bar and without channel clustering shows the smallest concentrations. In more detail, the concentrations are the highest close to the (imaginative) T-bar socket. In the case of all distances to the membrane, when the radius gets bigger, the concentrations decrease.

As in the case of the first peak of the 40 Hz stimulation considered before, we observe:

For a height close to the membrane, the difference of the concentrations in the case with and without T-bar, but clustered channels is approximately 25%. However, in the case of a height close to below the T-bar “roof,” which only is virtual in case of no T-bar, the difference is about 50%, i.e., the concentration in the case of the presence of a T-bar is about two times larger compared to the case without T-bar. For values of the radius farther away from the T-bar “socket” center, the values get more close.

In the case of not clustered channels and no T-bar, the values are much smaller for all considered cases, about an estimated factor of about 10 compared to the case of a T-bar and clustered channels, for all considered radii, and for all considered heights. In conclusion, the T-bar in combination with clustered channels is strongly superior to the case of no T-bar and clustered channels, and the case of no T-bar and not clustered channels even cannot compete with the other two cases.

So the calcium concentration behavior remains similar to the already discussed cases, also for ongoing action potentials, not only for the first peak. As well under variation of the frequency, at the peak moments, there are no major differences in the relations of the concentration relations compared to the first peak case we considered before.

With ongoing firing rate, we see a small synaptic depression caused by the calcium N-type channel behavior, that causes a rather small decrease in the peak size, which however has only a very minor influence on the quantitative results and no influence on the qualitative results for the comparison of the three geometry types. This small reduction of the calcium influx is due to VGCC gating depression effects.

#### 3.2.4. Sensitivity Analysis: Influence of Buffers and Pumps

**Figure 10:**
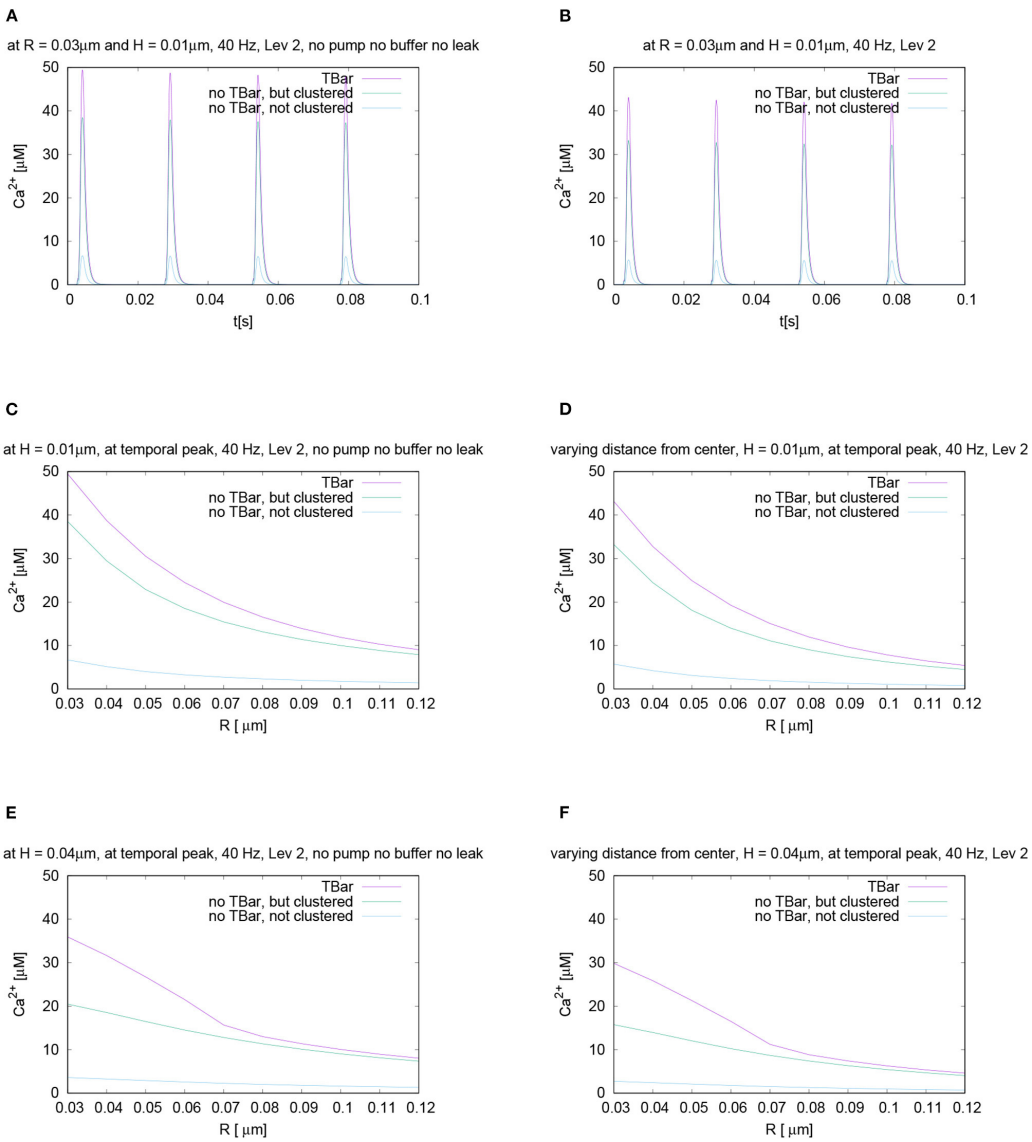
Compare the case of calcium microdomain concentrations in the presence of buffers and pumps with the case of the absence of buffers and pumps for all three geometric scenarios. Left column: Case without buffers and pumps, right column: standard case with buffers and pumps (to facilitate direct comparison, we repeat parts of Figures 6, 7). First row - **(A,B)**: Temporal evolution for fixed spatial point (i.e., concentration averaged over a circle), evaluation around the (imaginative in the case without T-bar) T-bar socket center at radius *R* = 0.03 and height *H* = 0.01: **(A)** without buffer and pumps, **(B)** with buffers and pumps. Second and third row - **(C–F)**: spatial profile at peak time. Evaluation of calcium concentration for *t* = 0.0042 s, at the influx peak, for different values of fixed height *H* and varying radius *R*, from the (imaginative in case of no T-bar) T-bar socket center. **(C,D)**: radius variation comparison for height *H* = 0.01μm [**(C)** without buffers and pumps, **(D)** with buffers and pumps], **(E,F)**: radius variation comparison for height *H* = 0.04μm [**(E)** without buffers and pumps, **(F)** with buffers and pumps]. At the second row—**(C,D)**—we are close to the membrane, at the third row–**(E,F)**—we are close to the (virtual) T-bar “roof”. Indeed, in all considered cases, the differences of the concentrations between the case with buffers and pumps and without them are comparably small. We observe that the absence of buffer and pumps causes the increase of the calcium concentration quantitatively, whereas the relative differences between the three anatomic cases do not change effectively.

To test for the influence of the buffer mechanism, the pumps, and the leakage, we performed the simulations switching off these effects. When comparing the three different anatomic cases, the results are very similar to the case with these effects, (cf. Figure 10) for the temporal and spatial profiles.

Even qualitatively, the calcium microdomain shapes show effectively the same behavior as in the case with buffer, pumps, and leakage. There is no major difference between the case of a large forward buffering constant and active pumps, and the case without buffering and without pumps.

#### 3.2.5. Variation of VGCC Number per AZ

Having varied different “software” parameters, we vary next to a “hardware” parameter: We vary the number of channels per AZ to test if the results we derived so far might depend upon the number of the channels at the AZ. We probe for if different channel numbers might cause a change in the relation of the concentration of the three geometric cases, the AZ with T-bar, the AZ without T-bar but clustered channels, and the case without T-bar and without clustered channels.

**Figure 11:**
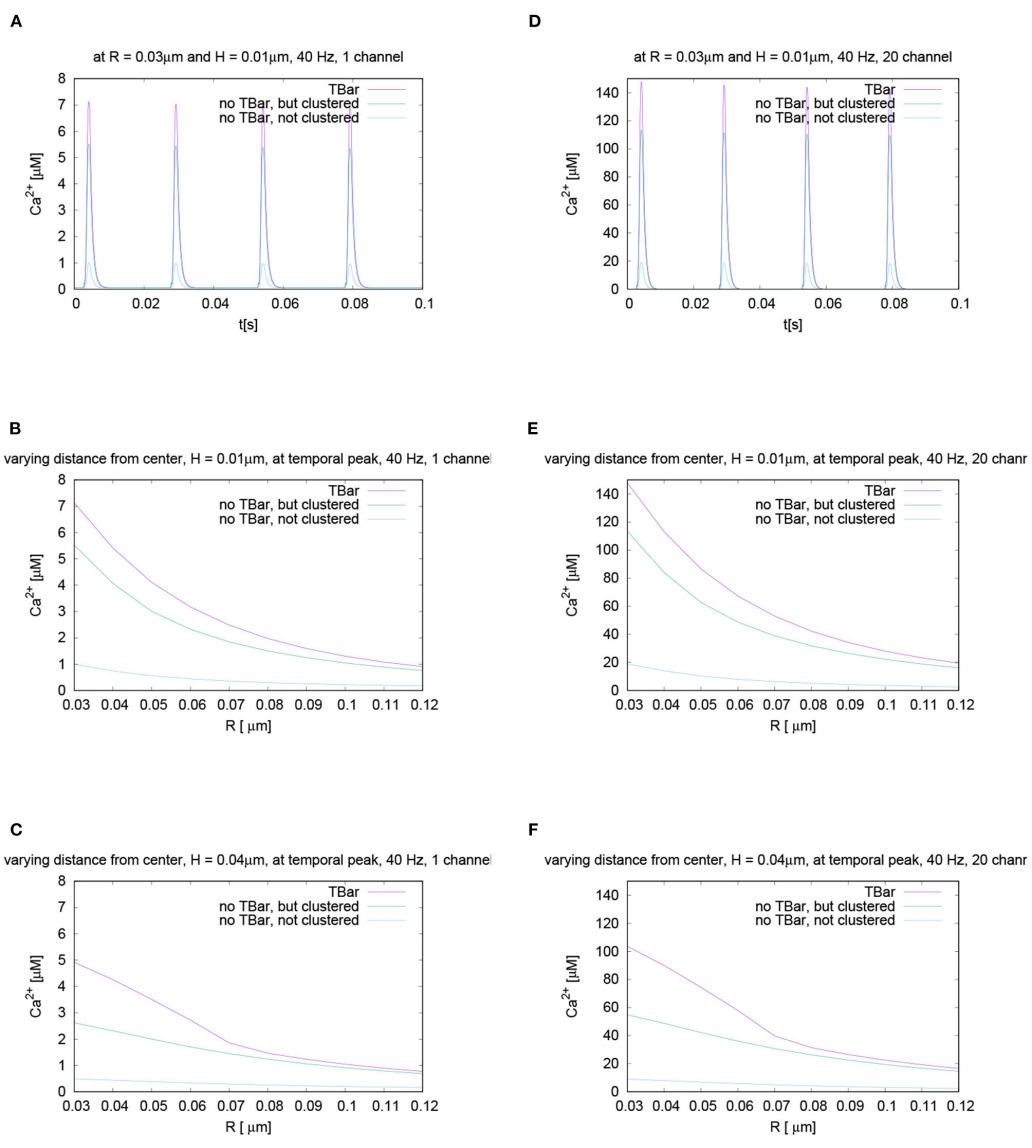
Sensitivity of results under variation of VGCC number, rest of parameters standard setup. Left column **(A–C)**: only one VGCC in the AZ, right column **(D–F)**: 20 VGCC in the AZ. First row-**(A,B)**: temporal evolution at fixed height and radius, second and third row-**(B,C,E,F)**: spatial profile at the first temporal peak, i.e., for a fixed time, but different values of height (second row close to the membrane, third row close to (virtual) T-bar “roof”) and varying radius. Note that the scale of the y-axis necessarily varies compared to the standard scale, but is equal for each given channel number graph. The scales of representation are quite different due to the different influx amounts caused by the strong difference in the number of VGCCs. Whereas the scales of the calcium microdomain concentration change, the relative differences between the different anatomic cases effectively remain the same as before. If we would omit the scale bar of the y-axis, it would be quasi impossible to distinguish between different VGCC numbers. The variation of the channel number in the AZ only has an influence on the scale of the calcium microdomain peak concentration, but the relations for the concentrations between the three anatomic cases remain practically constant.

For the VGCC number per AZ, we varied in a wide range (Nadkarni et al., 2010). Indeed, we probed for the following number of VGCCs per AZ: 1, 3, 6, 10, 20, 50, 100, and 200 VGCCs per AZ. Whereas, the graphics shown so far referred to the standard setup of 6 VGCCs, Figure 11 displays evaluations for the cases of 1 VGCC and 20 VGCCs per AZ.

We again show variations of the concentrations for the three cases over time for a fixed radius and a given height. As well, we evaluate the variation of the concentrations for a fixed time point at different heights, considering the variation of the radius with respect to the center of the (virtual) T-bar center. As the relations are the same as in all cases so far considered, we omit to repeat these results in an extended manner in the text, as they remain the same for all numbers of VGCCs. Whereas the scale changes, as more influx means more concentration, the relations do not change when comparing the three anatomic cases. The variation of the number of VGCCs has no major impact upon the relative influence of the T-bar upon calcium concentration compared to the case, where the T-bar is not present.

Quantitatively, an increase in VGCC numbers has a strong effect, but in a similar way for all three geometric cases: in all cases, we arrive at the result that the T-bar (clustered channels) has higher calcium microdomain concentrations, then follows clustered channels without T-bar, and at the end, lowest calcium microdomain concentration, the case of not clustered channels without T-bar. Indeed, the number of VGCCs is nearly proportional to the peak concentration of calcium.

The statement that the channel number is not important is due to the fact that we always compare the three geometric cases with each other for the *same* number of VGCCs. This is a qualitative comparison of fixed VGCC numbers.

Supplementary Figures S2–S6 display the cases for 3, 10, 50, 100, and 200 VGCCs. Indeed, the qualitative behavior is always the same: A change in the number of VGCCs just enhances the total scale of calcium microdomain concentrations, but not the relations which practically keep constant for all cases.

**Figure 12:**
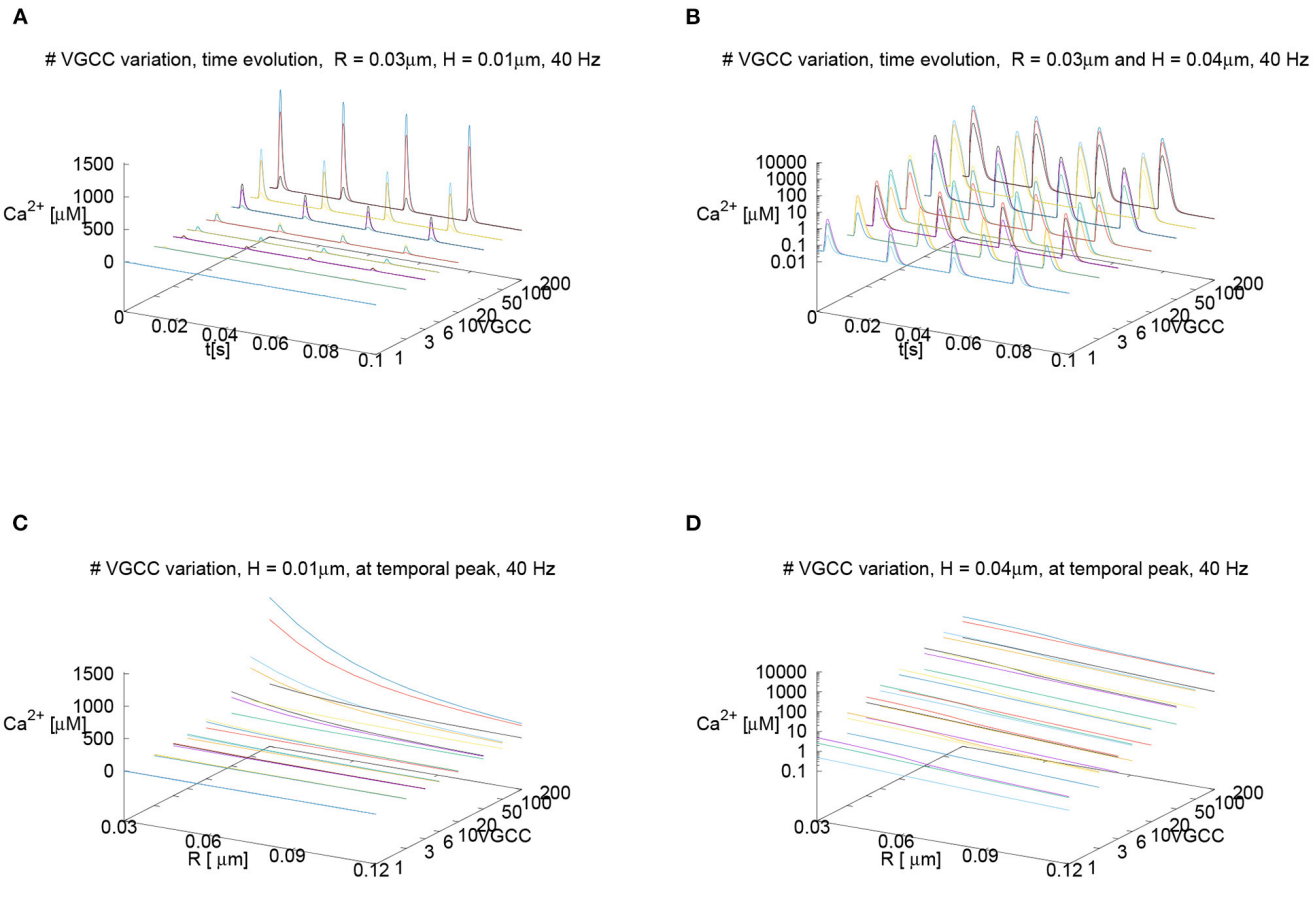
Displaying the calcium concentration profiles under variation of the number of calcium channels with the aid of 3D plots, where the number of channels represents the third dimension. Standard parameter set (besides channel number). In the upper row, we consider the time evolution of the calcium concentration at fixed spatial locations, in the lower row, we consider the spatial profile of the calcium concentration at the peak time and vary the radius of the evaluation for fixed height. In the left column, we consider a linear scale for the calcium concentrations, on the right column, we consider logarithmic scales for the calcium concentrations. VGCC number is always displayed with a logarithmic scale. **(A)** Time evolution for *R* = 0.03μm and *H* = 0.01μm, **(B)** Time evolution for *R* = 0.03μm and *H* = 0.04μm using a logarithmic scale for calcium concentration. **(C)** Spatial profile at peak time for variation of radius for *H* = 0.01μm. **(D)** Spatial profile at peak time for variation of radius for *H* = 0.04μm using logarithmic scale for calcium concentration. In all curves, we see clearly the nearly linear dependence of the concentrations upon the VGCC number. In particular, we see that independent of the channel number, for fixed channel number, the relation of concentrations between the three geometric cases is always practically the same—in particular, the logarithmic representation shows this fact in an impressing manner. The T-bar case is always substantially higher than the case without T-bar with clustered channels, and in the case when the T-bar is absent and the channels are not clustered, the concentration is even much smaller in all cases. Even the quantitative relations are always very similar: In case when we evaluate close to the membrane, for small height, the T-bar case shows about 25% elevated calcium concentration compared to the case without T-bar but channels still clustered, and in the case when we evaluate below the T-bar “roof”, the concentration is about a factor 2 bigger in the case with T-bar compared to the case without T-bar, but channels still clustered. In all cases, the concentrations are much smaller if there is no T-bar combined with a configuration where the channels are not clustered. Of course, the total influx is quasi proportional to the VGCC number, but this does not affect the relations for the three anatomic cases. Hence, the main message of our study is not sensitive to the variation of the VGCC number.

For the sake of completeness, we also display a comparison of channel number variation results with the aid of a 3D plot, (cf. Figure 12).

#### 3.2.6. Comparison of Averaged Calcium Concentration Below (Virtual) “Table”

Also, we compare the concentration profile for the averaged calcium concentration below the (virtual) table, i.e., within the computational subdomain U for the standard parameter choice. We observe a similar behavior as we had it before for the comparisons at the single points, but do not get new information. The comparison is displayed in the Supplementary Figure S7.

### 3.3. Numerical Robustness, the Dependence of Results Upon Chosen Grid Resolution

As we perform numerical computations based upon finite volume discretizations, one could ask if the results are numerically robust, or if they might, e.g., depend upon the computational grid we use or upon the grid refinement level. In order to be able to exclude that our results show grid dependent artifacts, we performed the analysis of our results by means of numerical grid convergence studies. For diffusion-reaction equations, results should become more and more stable with increasing grid refinement levels. To do so, we study the behavior of our results for the standard parameter set under grid level refinement. We study the case of the evaluation at a fixed spatial location over time, and we study the behavior in the case of the variation of the radius for a given peak and fixed height. For the latter case, we also compute the relative differences between the different levels for each given anatomical setup, and we see excellent numerical grid convergence. The differences to higher levels are below one per mill already for the standard refinement level 2 compared to levels 3 and 4. Therefore, we can conclude that our results are not dependent on our grid nor the grid resolution, and thus, our numerical results are highly reliable.

**Figure 13:**
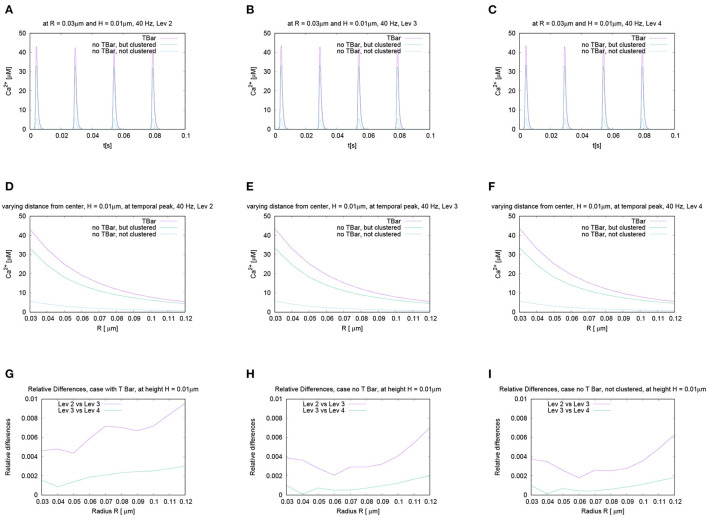
The aim of this figure is to demonstrate that the results we compute by means of vertex-centered finite volume methods are independent of the computational mesh, which is validated if they are independent of the grid refinement level. This means that we compare the results for making the grid finer and finer. If one can show this numerical grid convergence, one can trust the results. Here, we display results for the standard parameter set for different grid refinement levels: Standard spatial refinement level 2 as used for the results presented in the other figures, and spatial refinement levels 3 and 4. The first two rows display such a refinement test. The first row shows results for evaluations of calcium concentrations over time for a fixed height and radius, whereas the second row shows results for fixed time at the first peak, evaluated for a fixed height and varying radius. In the last row, we display the relative differences for the three different anatomic scenarios for the spatial profile. In detail: **(A–C)**: Calcium profiles for evaluation over time. The radius of calcium concentration evaluation around the (imaginative in the case without T-bar) T-bar socket center at radius *R* = 0.03 and height *H* = 0.01. Variation of grid refinement level, from level 2 to level 4. **(D–F)**: Evaluation of calcium concentration for *t* = 0.0042s, at the influx peak, for (for simplicity) one value of fixed height *H* and varying distance, radius *R*, from the (imaginative in case of no T-bar) T-bar socket center. Variation of grid refinement level, from level 2 to level 4. For the temporal **(A–C)** and the spatial profiles **(D–F)**, results are quite similar at all considered levels, also quantitatively. The visual comparison does not show significant differences. **(G–I)**: Relative differences of calcium concentrations along the radius at a fixed height, for all three geometric configurations. Comparing levels 2, 3, and 4. **(G)** with T-bar, **(H)** without T-bar but clustered channels, **(I)** without T-bar and no channel clustering. We see excellent numerical grid convergence for all cases. Our results are independent of the chosen grid resolution.

In detail: Numerical grid convergence tests. Figures 13A–F display numerical grid refinement tests of the results in the style we discussed before. Figures 13G–I compares the relative differences for the displayed values. We see excellent numerical grid convergence. Between levels 2 and 3, the relative differences are in the order of magnitude less than one per mill, for the case between levels 3 and 4, we are even in the region of about 0.1 per mill. Thus, our results are strongly reliable.

## 4. Discussion

In this study, we created a mathematical model describing calcium influx into the presynaptic active zone of the Drosophila NMJ for three different anatomical cases, namely for a geometry with T-bar, for a geometry without T-bar and clustered calcium channels, and for a geometry without T-bar and without clustered channels, i.e., with more broadly distributed channels.

Our *in silico* results quantify the influence of the T-bar and the channel clustering on the calcium concentration at the AZ.

This means that our results enable us to determine in a quantitative manner the efficacy of the T-bar anatomical structure upon presynaptic calcium concentration. In this study, we computed the calcium concentration in a fully spatio-temporal resolved manner (3 spatial dimensions plus one temporal one) for the Drosophila larval NMJ presynaptic AZ for those scenarios which are realized in biology, and we computed quantitatively the differences of the calcium concentrations at each point of the AZ.

Interestingly, the maximum values of the intracellular calcium microdomains close to the membrane/below the T-bar (as computed by us) are consistent with the values reported by Schneggenburger and Neher (2005).

### 4.1. Major Differences for the Three Geometric Scenarios

All simulations for all variation of parameters such as stimulation frequency or VGCC number have revealed that the calcium microdomain concentrations deploy the highest peak values in case of the presence of the T-bar diffusion obstacle, whereas in the case of an absence of the T-bar, the concentrations are substantially reduced, in particular, if the channels are not clustered. Given the experimentally observed fact that the T-bar comes in combination with clustered channels, and that without the T-bar, the channels are not clustered, the biological sense of the T-bar is clearly explained such that it is a major player to enhance calcium concentration strongly.

### 4.2. The Interplay of VGCC Calcium Influx and Pumps Plus Buffer Caused Reduction

The influence of the buffer is of minor importance for our results, as it has no impact on the qualitative results, and only a nearly negligible influence on the quantitative results. It seems that the calcium diffuses away from the point of influx and the AZ before the buffer can grab the major part upon the influx of the calcium to react with it in the AZ. This could mean that in the context of calcium microdomains at the AZ of the NMJ with and without T-bar, calcium diffusion and influx have a more significant effect on the concentration and shape of the calcium microdomain.

This observation in the context of calcium dynamics is consistent with the findings of Sneyd and Tsaneva-Atanasova (2003), where the authors have shown that in the case of calcium waves, the influence of buffers may be negligible under some conditions.

The influence of the number of VGCCs is quantitative, but not of qualitative order. We also assume that for more than 20 VGCCs, the calcium microdomain peak concentrations reach values, which do not seem to be very realistic anymore, which we use as a further hint to assume that the VGCC number at the NMJ AZs are located in the lower region of the values reported by Nadkarni et al. (2010), which refer to hippocampal neurons.

### 4.3. VGCC Depression

Our simulation results consider e.g., Figure 8D in the 100 Hz case, where it is best visible, show a small VGCC depression caused a reduction of the calcium influx at each action potential under repetitive action potential stimulation. This fits the general valid observation in the molecular neurosciences that calcium channels, in general, tend to calcium-dependent inactivation.

Indeed, there are a lot of mechanisms described in the literature that account for calcium channel inactivation in many cases, but also activation in some circumstances (Lee et al., 2000; Thaler et al., 2004). Such mechanisms might be the interaction of the channels with buffers, specific signaling molecules or proteins, or exons.

If the VGCC depression would be stronger due to e.g., the presence of proteins that tend to block the channels, the reduction of the calcium influx would be stronger, such that at each following action potential, the peak of the influx would be smaller.

However, very likely, this would not affect the validity of the results derived here, because this influx reduction would affect all three anatomical cases we discussed in this study in a similar manner. After all parameter studies we performed, the relation of the peak concentrations for the case with and without T-bar remained similar. In particular, we have demonstrated this property by means of varying the VGCC number, which resulted in a large difference in scale of the total influx, where however the relations of total peak concentrations between the different anatomical cases remained effectively the same. Therefore, we can conclude that also with stronger reduced calcium influx, our statement that the T-bar obstacle enhances calcium concentration remains unchanged also in case of stronger calcium-dependent VGCC inactivation.

Finally, calcium-dependent channel inactivation effects happen at a significantly smaller scale than the effects considered within our study. We are using the homogenized model developed by Borg-Graham (1998), where the parameters used were fitted to typical cases. It would be outside the scope of this study to change the parameters of the model of Borg-Graham (1998). Anyhow, this is not of the major importance of our results, as all sensitivity analysis studies which we have performed have shown that the influence of the T-bar obstacle upon calcium peak concentrations is such stable that this very likely also will not change at all if other calcium influx models would be used such as models where the VGCC depression would be enhanced compared to the model of Borg-Graham (1998).

Note that the effect of small peak reduction with ongoing firing rate due to synaptic channel depression is not the synaptic plasticity we mean in this study, and we think this small effect has nothing to do with synaptic plasticity.

### 4.4. Synaptic Plasticity and T-Bar

Indeed, the application of the terminus “synaptic plasticity” might be considered as ambiguous, but is getting clear based on our definition we mean in this context:

When talking about plasticity effects of the T-bar, we mean that the presence (after growth) of this obstacle has a substantial impact upon calcium microdomain concentrations, i.e., the synaptic plasticity we mean refers to the difference of the presence and absence of the T-bar.

Synaptic plasticity in our context means to compare the synaptic behavior of the three different geometric types, i.e., to evaluate the influence on synaptic effects in the case with and without T-bar and in the case of clustered and not clustered channels. In this sense, the T-bar causes the plasticity effect to enhance calcium concentration.

However, we do not model the growth of the T-bar itself but compare the synaptic behavior for the three cases. In this sense, the T-bar has a measurable effect upon synaptic plasticity, but not at the time scale at which we compute, but when comparing the result of a synapse with the grown T-bar with a synapse where no T-bar is present.

A major question of Wichmann and Sigrist (2010) is if the T-bar can be considered as some sort of “plasticity module” related to synaptic transmission strength (Wichmann and Sigrist, 2010) (cf. namely the concluding section of the review). Indeed, as the results we present in this study show that the T-bar anatomical obstacle strongly enhances the calcium microdomain concentration at the active zone, our *in silico* study shows the impact of the T-bar anatomical obstacle upon vesicle release probability (given that enhanced calcium concentration enhances vesicle release probability). Therefore, the T-bar has a strong impact on plasticity in the sense that once grown, vesicle release probability is enhanced due to enhanced calcium microdomain concentrations, and thus, those synapses which harbor a T-bar likely show substantially enhanced synaptic transmission of action potentials.

In this sense, we have revealed why and in which sense the T-bar is a “plasticity module,” as stated in the review by Wichmann and Sigrist (2010). Of course, it might be the case that the T-bar harbors additional plasticity effects not discussed in this study.

Note that in this approach, we consider comparably short intervals of action potential stimulation and calcium influx, as the major question of our study is to relate the anatomical structure of the T-bar to the calcium concentration, comparing the case of AZs with and without T-bar. Our study does not consider long-term comparisons, as they would ask for additional effects such as major changes in calcium influx, which might differ for the three cases. However, in such a case, it would not be possible anymore to study the question of the influence of the anatomical structure of the T-bar in a way independent of additional effects.

### 4.5. Calcium Concentration and Vesicle Dynamics

#### 4.5.1. Biophysical Explanation of Enhanced Release Probability

The T-bar has a significant influence on the intracellular calcium concentrations close to the AZ and hence has a substantial influence upon the release probability of mature vesicles if those are present close to the AZ.

If the vesicle is located farther away from the membrane, but “below” the T-bar, the T-bar obstacle substantially increases the calcium concentration (even more than close to the membrane) and, thus, establishes a substantially enhanced release probability also for vesicles not directly located at the membrane - even compared to the case of clustered channels but absent T-bar. Thus, clustering of channels alone enhances release probability close to the membrane, but the T-bar obstacle makes a major difference in particular for vesicles not directly located at the membrane, but “below” the “roof” of the obstacle of the T-bar.

As the T-bar causes higher calcium concentration, it causes also vesicles not directly located at the membrane to have a strongly enhanced release probability compared even to the case of clustered channels, but no T-bar. The difference for the case with and without T-bar might decide if a vesicle either will be exocytosed or not.

Indeed, our statement that the T-bar AZ enhances calcium concentration and vesicle release probability fits very well with the experimentally observed fact that synapses with several T-bars show permanent enhanced vesicle release, as stated by Wichmann and Sigrist (2010) (page 9), and it explains this observation by means of biophysical arguments.

Furthermore, the enhanced calcium concentration, in the case of a T-bar, as shown by our simulations, explains why high release probability synapses are synapses that harbor T-bars, but low-release probabilities are those synapses without T-bars, as also assumed by Wichmann and Sigrist (2010) (page 9).

Our study explains in a biophysical manner *why* T-bars enhance release probability, whereas pure biological experiments only were able to show *that* synaptic strength strongly depends upon the presence or absence of a T-bar (Wichmann and Sigrist, 2010) (page 10).

These biophysical well-founded conclusions also allow us to understand why the BRP mutants, where the calcium channel clustering is disturbed and which do not have a T-bar deploy reduced calcium influx and low release probability (Goel et al., 2019a) - the lack of the T-bar and the lack of calcium channel clustering causes that the calcium microdomain concentration is much smaller. Thus, one might even speculate if the calcium influx itself is not much smaller, but as the calcium distributes much faster without T-bar, it has much fewer effects upon vesicle release probability due to the strongly reduced peak concentration at the presynaptic AZ, as our study has revealed. Having shown in our results that the T-bar diffusion obstacle has an important impact upon the calcium microdomain concentration, the question of why BRP mutants that lack a T-bar deploy lower release probability is answered by our results at least in major part, even though our model does not incorporate the T-bar size dependent growth and clustering of channels, but considers fixed scenarios at this stage.

#### 4.5.2. Possible Extension: Combine Calcium and Vesicles

As experimental studies have shown (Graf et al., 2009; Goel et al., 2019a), calcium channel numbers and clustering are correlated with the size of the T-bar. As we do not consider channel and T-bar growth, but moreover compare already existing anatomical states, the effect cannot be explained with the aid of our simulations. However, this very interesting experimental observation could be the object of future *in silico* studies based upon our framework.

In particular, our framework and even the data presented in this study could be used to compute release probabilities for vesicles based on calcium concentrations, depending on the geometric context, i.e., to relate the form and function of synapse structures. Such evaluations could be done based on our given data set by means of combining them with available calcium release probability models. Indeed, we plan such applications of our data and our framework for future studies, also in the interplay with the vesicles.

In the middle run, it would be an interesting task for future studies to combine calcium simulations as performed in this study with vesicle dynamics computations. This combination (incorporating also interesting information such as given in Gonzalez-Bellido et al., 2009) would complement our former study concerning the dynamics of the vesicles of the Drosophila larval NMJ (Knodel et al., 2014) which we will discuss in more detail in the next section in order to evaluate if this study might help further understanding of the results derived within our former study, cf. Section 4.6.

Such a model combining calcium concentration dynamics and vesicle dynamics might facilitate understanding in more detail how and why the release probability also depends upon the spatial distance between the docking vesicles, and the impact upon endocytosis efficiency. While at the present stage, such questions are beyond the scope of the present study; however, a future model and simulation approach might also help to get further insight into the different basic mechanisms between tonic and phasic synapses (Atwood et al., 1993; Xing and Wu, 2018a).

### 4.6. Putative Impact of T-Bar Effects Upon Different Bouton Type Characteristics

#### 4.6.1. A Former Computational Study of Vesicle Dynamics and Bouton Types

In a former study, we investigated the relation of form and function of the different bouton types of the Drosophila larval NMJ, which appear in this context (Knodel et al., 2014). We developed a mathematical model and performed simulations, which demonstrated that our model fitted quite well to the experimental data. In this former study, the vesicular exocytosis and dynamics of the whole NMJ bouton with several AZs were modeled. In particular, our model and simulations enabled us to unravel the relation of form and function of the differently sized bouton types at the Drosophila NMJ presynaptic boutons on the base of our *in silico* studies, and our *in silico* prediction was validated by the experimental data.

In detail, the basic question of the former article was to understand why the NMJ harbors two different bouton types, namely the small 1s boutons and the big 1b boutons (Johansen et al., 1989; Atwood et al., 1993).

The restriction to 1s and 1b boutons was due to consideration of experiments performed at abdominal muscles 6 and 7, which are exclusively innervated by 1s and 1b boutons. Bouton 1s and 1b have glutamatergic synapses. Among other muscle segments, namely muscles 12 and 13, there exists an additional bouton type called type II. Type II bouton harbors aminergic synapses. Type 1s boutons and type II boutons show phasic, while type 1b show tonic behavior.

Assuming a diffusion process describing vesicle movement within the 3D boutons, we developed a mathematical model of spatio-temporal resolved vesicle dynamics and applied this model to different sized boutons which aimed for 1s and 1b boutons with different numbers of synapses as realized typically in the NMJ. The model approach of our former study simulated vesicle dynamics within complete single presynaptic boutons. A major element of the evaluations was to apply sensitivity analysis upon bouton size, vesicle release probability and active zone number. These studies allowed us to unveil that high vesicle release probabilities allow for strong vesicle release and thus high EPSPs quasi independent of the bouton size at the beginning of the action potential stimulation. However, our investigations showed as well that the high support in case of high release probability drops down rapidly independent of the bouton size. Moreover, we showed that large boutons allow for long-term support of vesicle release quasi independent of the release probability due to the interplay of release probability and available vesicles. Therefore, we predicted that the NMJ is constructed such that the small 1s boutons are equipped with high release probability sites, and the large 1b boutons are equipped with low release probability sites. The experimental data, which were a complementary part of our study (Knodel et al., 2014) (derived by our experimental partners at this stage) verified our predictions. So our *in silico* approach enabled us to understand the relation of the bouton size with the different action potential patterns which the brain imposes to the different bouton types. Our study enabled to understand why and how the Drosophila larval NMJ combines different sized boutons. We figured out that the combination of sizes and release probabilities we proposed allows the larval movement using a very efficient technique of neuronal plasticity.

Whereas, our former study proposed that the 1s and 1b boutons have different release probability patterns leading to different plasticity patterns (long-term low level EPSPs for the 1b bouton due to low release probability combined with huge size of the ready-releasable-pool vs. short-term high level EPSPs for the 1s boutons due to high release probability leading to fast depletion of the ready-for-releasable pool, which, however, would be similar also in a bigger bouton, so a small bouton suffices for strong short-term EPSPs), we did not speculate on the question of the reason of these different patterns.

#### 4.6.2. Speculative Relation of T-Bar AZs and Bouton Type

Independent experimental studies by Kurdyak et al. (1994) revealed that 1s and 1b boutons show different plasticity dynamics, 1b show tonic and 1b and type II boutons phasic behavior. In addition, more recent studies have shown that the different bouton types show also different calcium dynamics behavior (Xing and Wu, 2018a,b) such that namely 1s and type II boutons show enhanced calcium dynamics compared to the larger 1b boutons. These results, in combination with our former study, pose the question if different calcium dynamics of the two bouton types might be in relation to different release probabilities, as calcium concentration is assumed to enhance release probability. Moreover, it is tempting to ask if this study might give additional hints for some of the reasons for the different release probabilities of the different bouton types.

In particular, it is tempting to ask for the reason for the different calcium profiles. One possible solution might be that different anatomical structures at the AZs would be responsible for different calcium microdomain concentrations leading to different release probabilities, which coincides with the before already described challenge to understand the role of the T-bar in the AZ and its putative impact upon calcium concentration profiles. Indeed, in this study, we investigated the relation of form and function at a slightly smaller scale compared to our former study of vesicle dynamics at the scale of complete boutons. Moreover, instead of considering vesicle dynamics, we considered the dynamics of calcium, which underlies vesicle dynamics. A challenging question is if it might be possible to combine the results of both model simulation studies, this study and the former study (Knodel et al., 2014).

Therefore, it would be tempting to speculate if the difference in release probability between 1s and 1b boutons might have its reason in enhanced numbers of T-bars in the case of 1s and type II boutons such that the enhanced number of T-bars might be responsible for enhanced calcium dynamics in 1s and type II boutons compared to 1b boutons.

Indeed (Xing and Wu, 2018a) stated that (He et al., 2009; Lu et al., 2016) have shown that the AZ density in 1s and type II boutons is higher than in 1b boutons. More in detail, Lu et al. (2016), have counted the AZ numbers (with and without T-bar) in 1s and 1b boutons, but to our reading their counting did not distinguish between the AZs with and without T-bars. However, among other results, they found that 1s boutons have less AZs than 1b boutons but release more neurotransmitters, and deploy higher calcium transients due to much higher calcium entry per volume area. He et al. (2009) found that the calcium transients in 1s boutons had higher amplitude than those in 1b boutons, and longer time decay constant. Moreover, He et al. (2009) stated that a reason for the enhanced calcium profile of the 1s boutons compared to the 1b boutons seems to be related to the fact that 1s boutons have a 63% higher number of active zones per bouton volume than 1b boutons.

Despite the fact that (He et al., 2009; Lu et al., 2016) show several factors which influence the higher calcium dynamics in 1s and type II boutons, to our knowledge and reading, so far there exists no statistics for 1s, 1b and type II boutons comparing the number of AZs with T-bars with the number of active zones without T-bars distinguished by the bouton type. Also, we are not aware of experimental statistics referring to the question if e.g., the number of calcium channels per AZ differs between different bouton types, as also this factor might have an important influence upon the comparison.

Therefore, the results of our study open a perspective if maybe the relation of AZs with and without T-bar might be different between the different bouton types of the NMJ.

#### 4.6.3. Restrictions of Hypothesis

However, we are quite careful with the speculation of some sort of relation of T-bar number and bouton type. Explicitly, we name our idea an hypothesis but not any kind of result of our study.

It is very important to keep into account that in this study, we always compared the T-bar anatomy with the case of no T-bar always using the same parameters concerning other effects. However, when comparing 1s, 1b, and type II boutons, presumably, this method cannot be applied. Presumably, the surrounding parameters are different for different bouton types, as reported e.g., in Xing and Wu (2018a,b), where the authors have shown that the calcium levels and clearance mechanisms likely are different between the different bouton types.

Besides the fact that this article demonstrated that the hypothesis of our former study (Knodel et al., 2014) is true (that 1s boutons have a high release probability for strong short term vesicle supply, but 1b boutons have low release probability allowing for weak but long term supply of vesicles), we have to conclude that inside a *given* bouton of the specified type, the T-bar presence makes a major difference at a single AZ.

However, only in case, a statistical counting of T-bars in the different bouton types would reveal that the high release probability boutons have more T-bars, then the results of our study would imply that these T-bars likely are a major factor of the different firing patterns of the different bouton types.

So while our study is able to unveil the sense of the T-bar in an AZ compared to the case that the T-bar is absent and, thus, gives important insight into the biological reason of the T-bar growth, we have to restrict ourselves: With those experimental data known to us so far combined with the results of this study, it is not possible to give any kind of well-founded answer to the question why 1s boutons and type II boutons show higher release probability than 1b boutons. While we dare the hypothesis that maybe the relative number of T-bars might be enhanced in phasic boutons (1s, type II) of the NMJ compared to the 1b case, we do not claim that this hypothesis is safe at all. The conditions and parameters might vary strongly between the different bouton types.

An additional possibility would be further to speculate if the T-bars in case of the 1s boutons and type II boutons are bigger compared to the 1b case, but also this assumption is highly speculative and not any kind of result of this study. We are not aware of any kind of relevant experimental data in this direction.

## 5. Conclusion

Exploiting the results of the simulations of our model, in this study, we elaborated the relation of form and function and the biological sense of the T-bar anatomical structure at the Drosophila neuromuscular junction presynaptic AZ, and this study gives concise answers to at least a part of the major questions asked in the review (Wichmann and Sigrist, 2010), as discussed namely in Sections 4.4 and 4.5 of this paper.

Indeed, our study gives a very simple answer to the question asked in the title of the review (Wichmann and Sigrist, 2010): “The Active Zone T-Bar — A Plasticity Module?” The answer given by our study is yes in the sense that the presence of the T-bar causes a substantially enhanced calcium microdomain concentration profile compared to the case without T-bar and, thus, strongly influences vesicle release probability. Our results do not imply a short-term plasticity effect, but the plasticity effect refers to the comparison of the case before when a T-bar is grown, and once when it is grown. The anatomic form of the T-bar of the AZ of the synapses of the Drosophila larval NMJ has a strong influence on the calcium concentration. The enhanced calcium concentration in the case of a T-bar increases vesicle release probability. The form impacts the function of this presynaptic AZ. This means that our study has revealed the relation of form and function of the T-bar in the presynaptic active zone of the Drosophila larval NMJ.

Interestingly, we found this answer by means of a purely *in silico* study.

Our investigations in this study motivate experimental research to count and compare the AZs with and without T-bar to compare the relations for the different bouton types of the NMJ in order to evaluate if maybe the portion of AZs with and without T-bar might influence if a bouton type is a tonic or phasic, but this speculation is not a result of this study and cannot give a definitive answer to this question at this stage.

As detailed investigations have shown that the T-bar shape is more complex than just a simple “umbrella” (Wichmann and Sigrist, 2010), it would be interesting to perform simulations in the case of realistic reconstructed geometries. Such computations are possible with our simulation framework UG4. Therefore, we plan to extend our computations presented here to realistic reconstructed T-bar geometries within future studies.

We intend to apply our framework and our data to study in more detail the relation between the form of synapses and vesicle release probability caused by different calcium concentration profiles in different geometric scenarios, also in the case of other synapse types.

The review of Wichmann and Sigrist (2010) gives examples of other synapses also of other animals, where similar anatomical structures exist at synaptic AZs, so our investigations refer to a rather general field of studies that aim to unveil casual structure-function-relationships.

In conclusion, our framework and methods complement given research published by other groups, and our methods and results might stimulate further research for the relation of form and function at synapses, concerning calcium dynamics and their influence on vesicle release probabilities, and help develop a detailed understanding of the relation of form and function for neuronal processes.

## Data Availability Statement

The original contributions presented in the study are included in the article/Supplementary Material, further inquiries can be directed to the corresponding author.

## Author Contributions

MK and GW developed the model. MK and RD determined the model parameters based on the literature. MK performed the simulations and performed the evaluation of the results with the support of RD. MK, RD, and GW wrote the manuscript. All authors contributed to the article and approved the submitted version.

## Funding

This work has been supported by the German Ministry of Economics and Technology (BMWi) in the project HYMNE (02E11809B).

## Conflict of Interest

The authors declare that the research was conducted in the absence of any commercial or financial relationships that could be construed as a potential conflict of interest.

## Publisher's Note

All claims expressed in this article are solely those of the authors and do not necessarily represent those of their affiliated organizations, or those of the publisher, the editors and the reviewers. Any product that may be evaluated in this article, or claim that may be made by its manufacturer, is not guaranteed or endorsed by the publisher.

## Footnotes

^1^Note that we use the words “flux” and “current” like a synonym, as mathematically, we have a flux, whereas biophysically, this corresponds to a current.

## Supplemental Data

The supplemental material displays simulation movies of calcium dynamics influx into the presynaptic AZ of the Drosophila larval NMJ, for the case of anatomy with T-bar, and anatomy without T-bar but clustered channels, and anatomy without T-bar and channels not clustered. Buffer concentration simulation movies are attached as well for the three geometric cases. Simulations fully spatio-temporally resolved, 3D in space plus time. The simulation domain opened by means of a cut plane. Simulations displayed for the case where the major part of the computational domain (as opened by means of a cut plane) is shown, and also for the case where the AZ around the T-bar (and corresponding regions for the other two geometric scenarios without T-bar, i.e., with and without clustered channels) are zoomed. Furthermore, a supplemental file displays several figures to which we refer in the text of the article itself as additional information and a list of abbreviations. For the details of the movie type (anatomical structure, perspective, concentration type), we refer to the table of section 1 “SUPPLEMENTAL MOVIE DESCRIPTION” of the supplementary text file.
