# Supplementary material for: Influence of T-Bar on Calcium Concentration Impacting Release Probability
Source: Front Comput Neurosci. 2022 May 2;16:855746. doi: 10.3389/fncom.2022.855746 (PMC9108211; doi:10.3389/fncom.2022.855746)
Supplement: Supplementary file 1 [file Data_Sheet_1.pdf]

Supplementary Material: Influence of T Bar on calcium concentration impacting release probability

Markus M. Knodel [1,*], Ranjita Dutta Roy[2], and Gabriel Wittum [1,3]

[1] Goethe Center for Scientific Computing (GCSC), Goethe Universität Frankfurt, Frankfurt am Main, Federal Republic of Germany
[2]Science for Life Laboratory, KTH Stockholm, Sweden,
[3] Applied Mathematics and Computational Science, Computer, Electrical and Mathematical Science and Engineering Division, King Abdullah University of Science and Technology, KAUST, Thuwal, Saudi Arabia
* markus.knodel@gcsc.uni-frankfurt.de

1 SUPPLEMENTAL MOVIE DESCRIPTION

The supplemental movies V1-V9 refer to the following simulation setup and the corresponding figures of the paper:

Video	Figure (paper)	Concentration	T-bar present	Channels clustered	Zoomed view
1	5 A	calcium	yes	yes	yes
2	5 B	calcium	no	yes	yes
3	5 C	calcium	no	no	yes
4	4 A	calcium	yes	yes	no
5	4 B	buffer	yes	yes	no
6	4 C	calcium	no	yes	no
7	4 D	buffer	no	yes	no
8	4 E	calcium	no	no	no
9	4 F	buffer	no	no	no

2 SUPPLEMENTARY FIGURES

The following figures show different details of the simulations, which are indicated in their respective caption. Namely, we show the shape of an action potential, and graphics for the calcium concentrations under variation of the VGCC numbers, and concentration profiles averaged over the geometric zone below the (virtual) T-bar "roof".

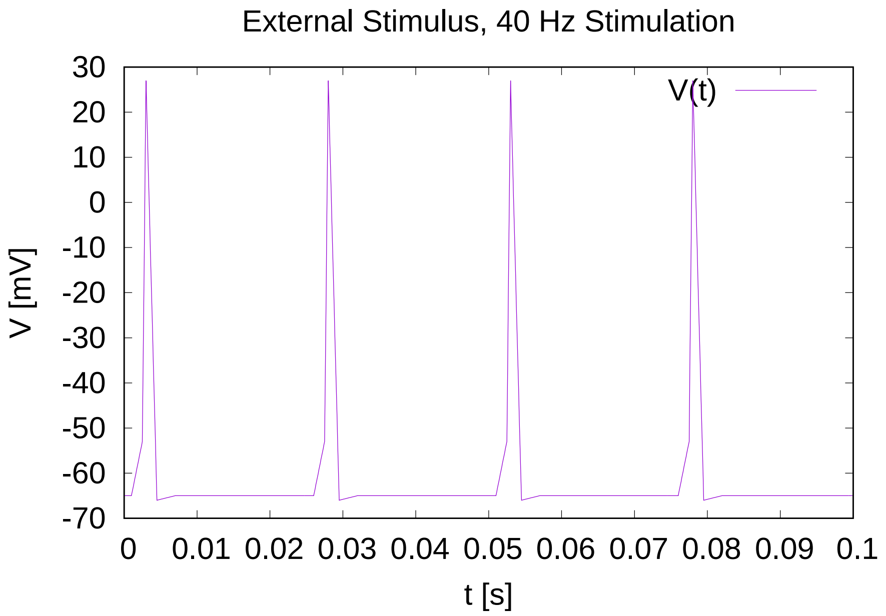

Figure S1. Imposed action potential stimulus shape at synapse membrane for the case of 40 Hz.

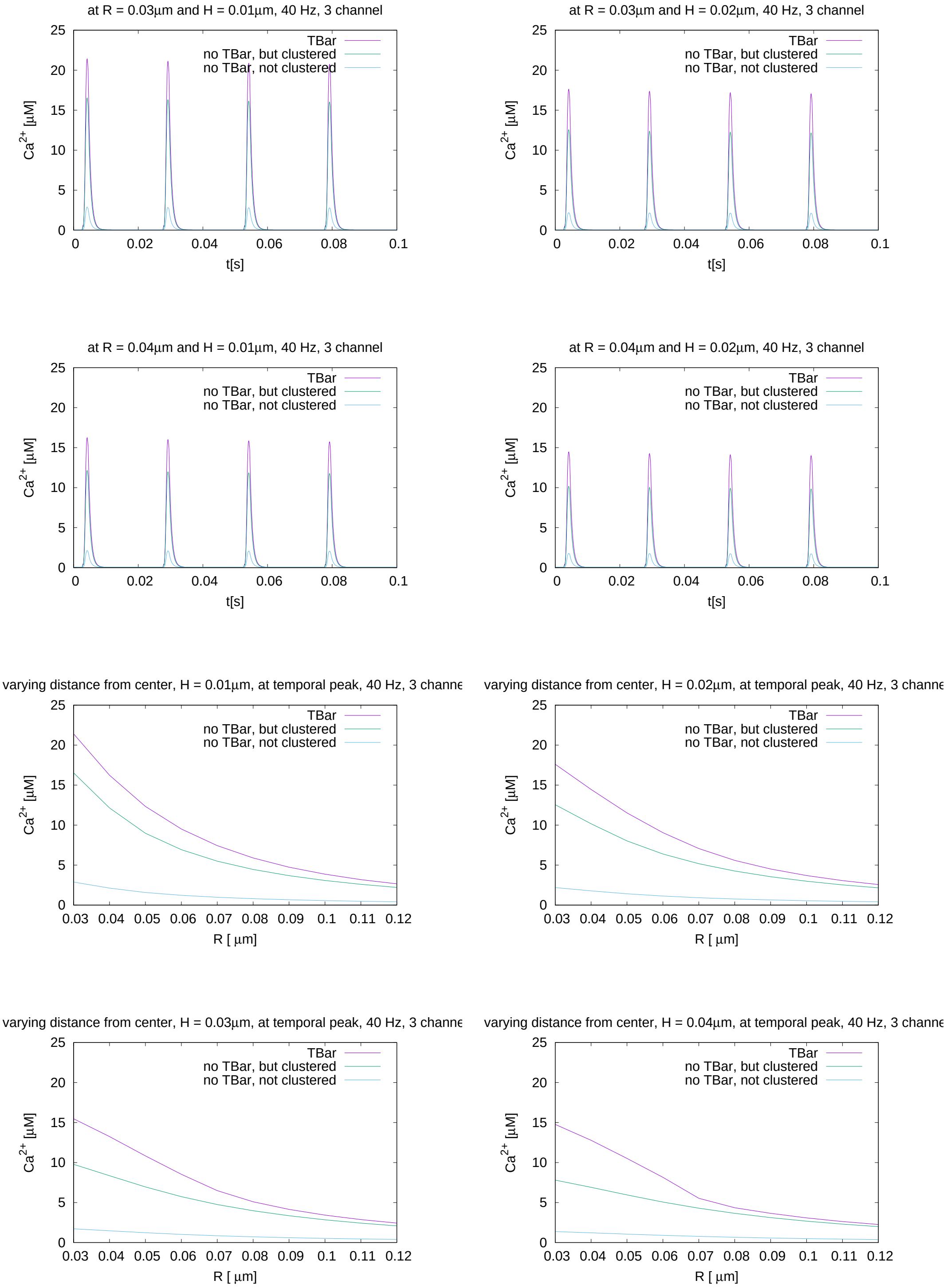

Figure S2. Variation of channel number: assuming 3 VGCCs per active zone, other parameters standard set.

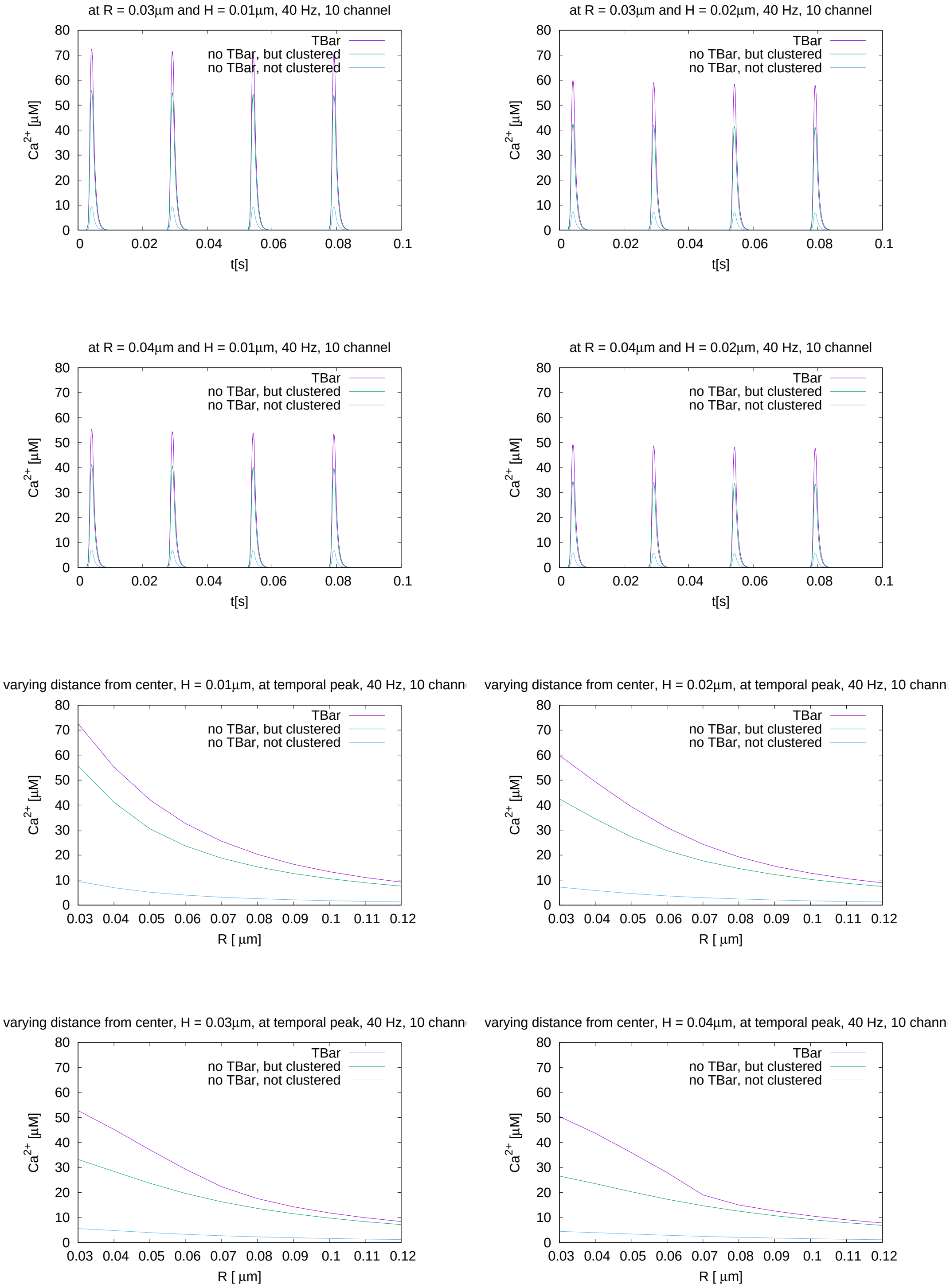

Figure S3. Variation of channel number: assuming 10 VGCCs per active zone, other parameters standard set. Note that the scale of the y axis necessarily varies compared to the case of other channel numbers, but is equal for each given channel number graphs.

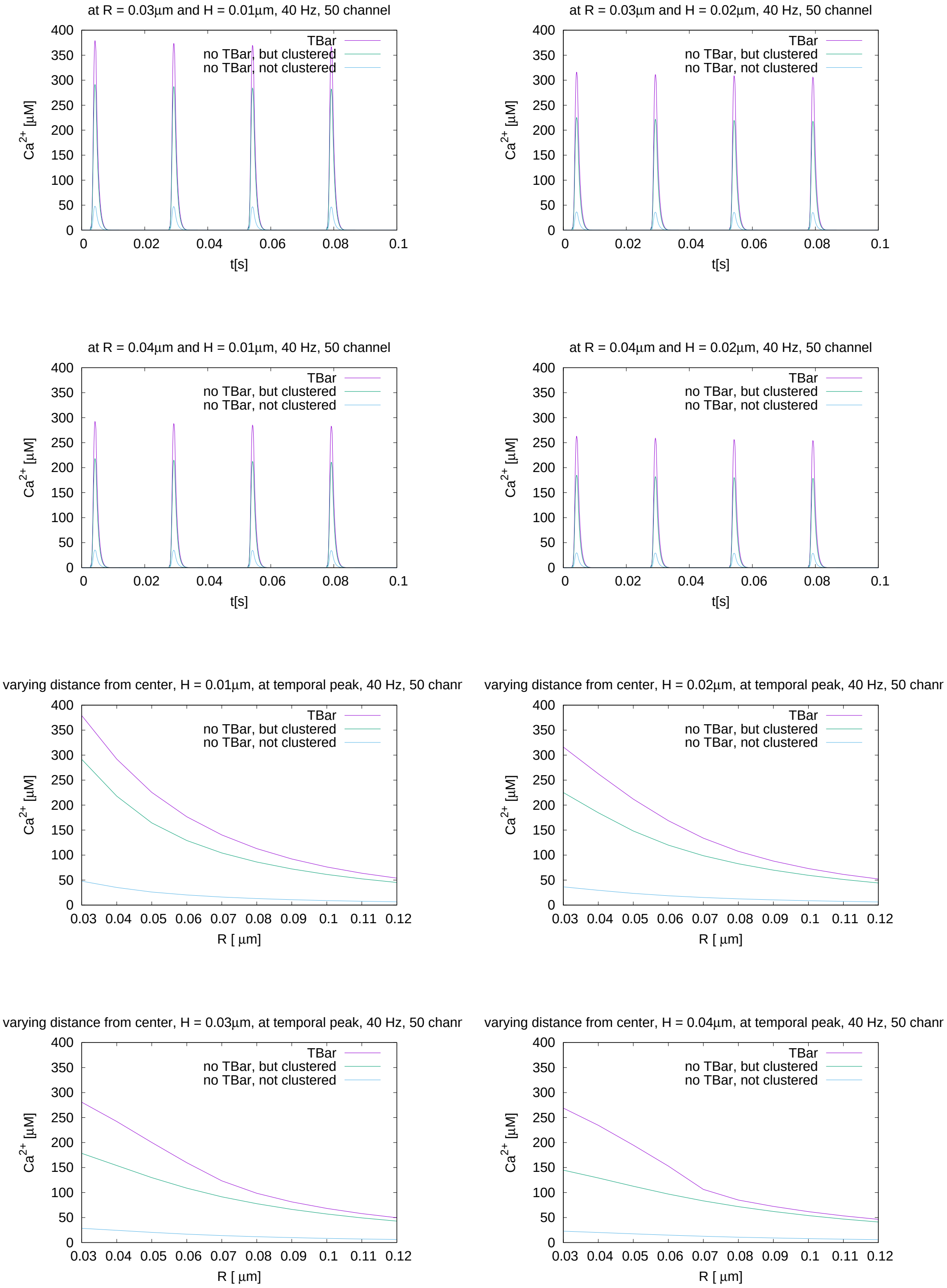

Figure S4. Variation of channel number: assuming 50 VGCCs per active zone, other parameters standard set.

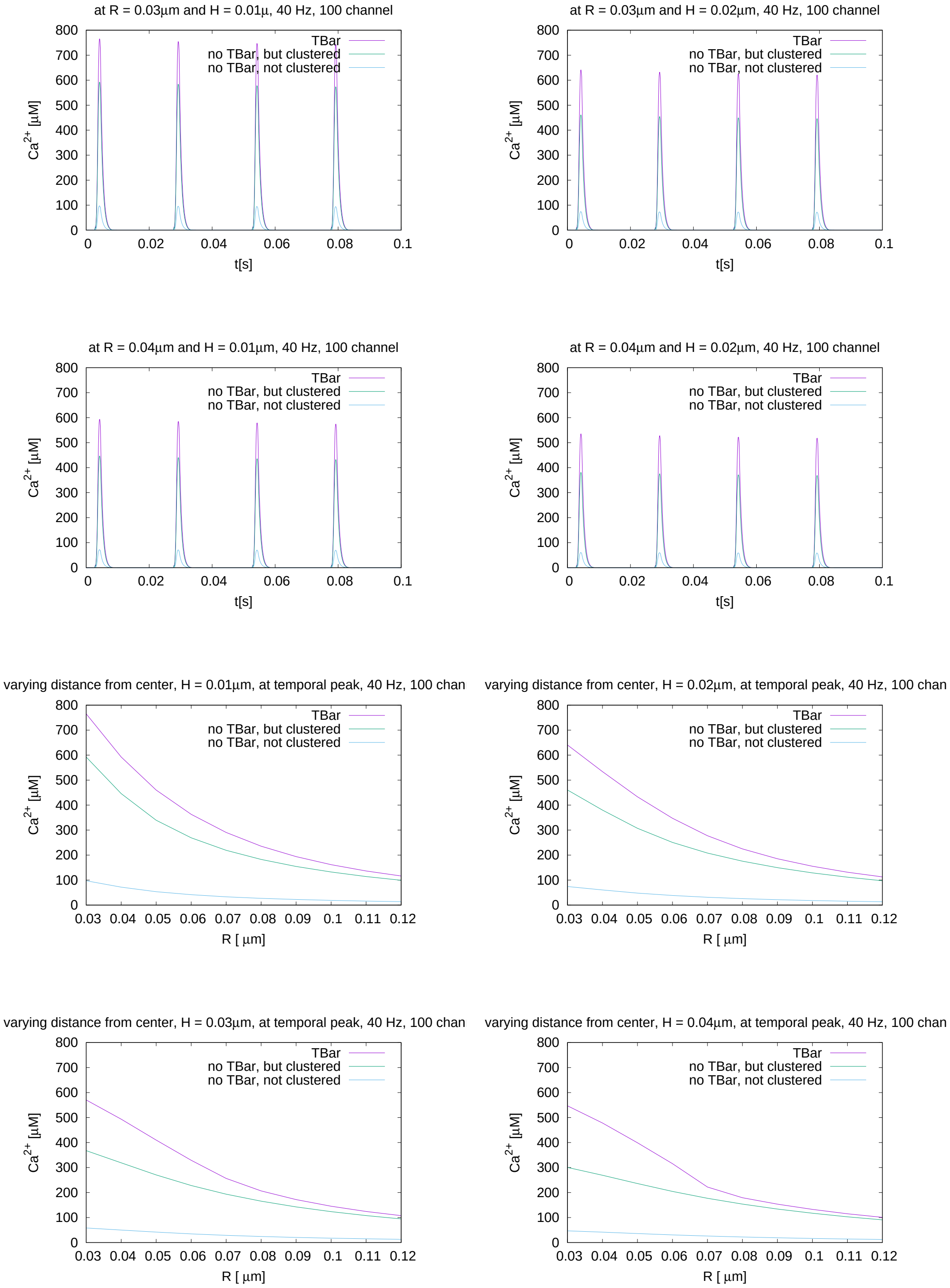

Figure S5. Variation of channel number, assuming 100 VGCCs Other parameters standard set.

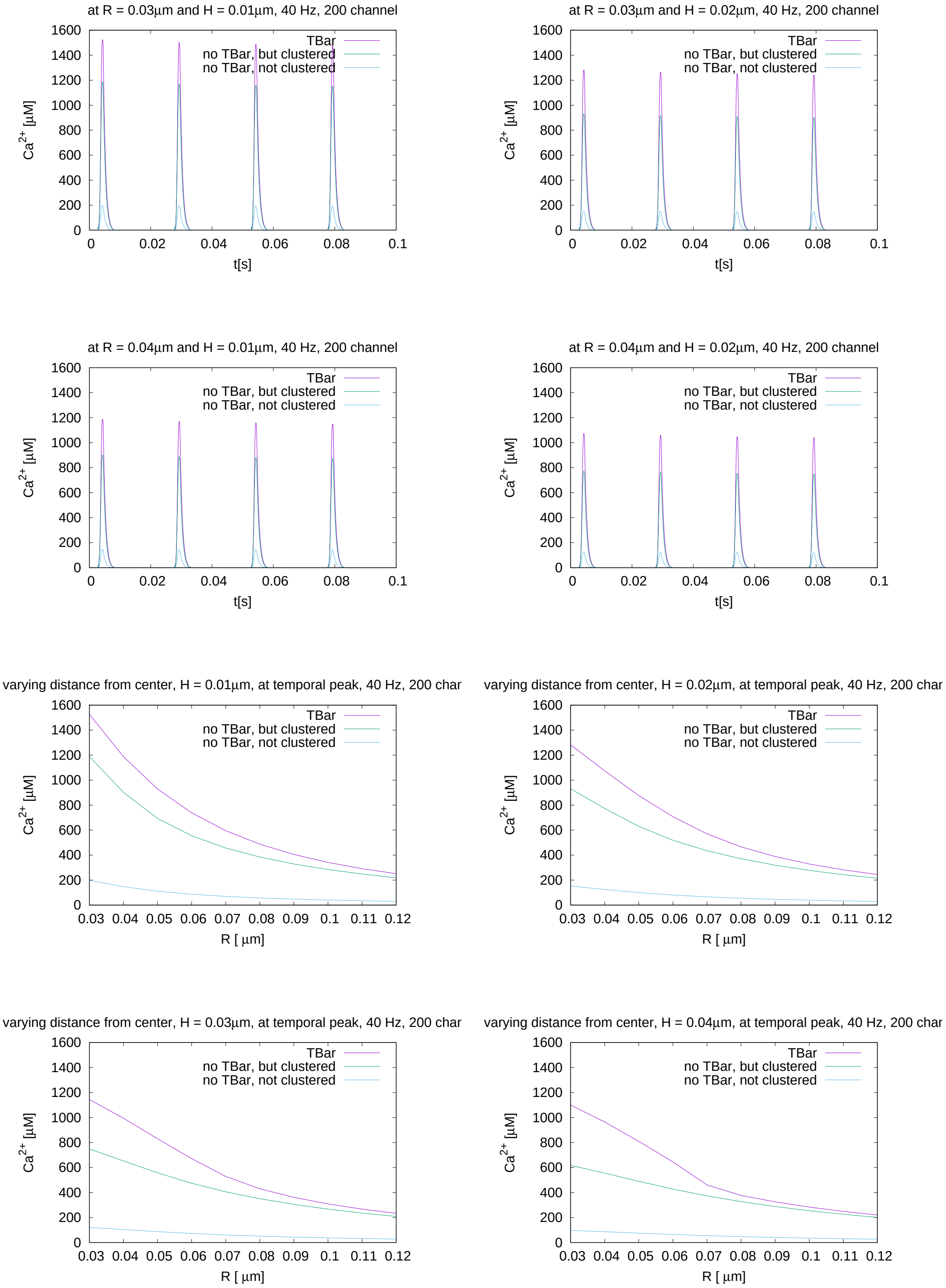

Figure S6. Variation of channel number, assuming 200 VGCCs (presumably extremely too much). Other parameters standard set.

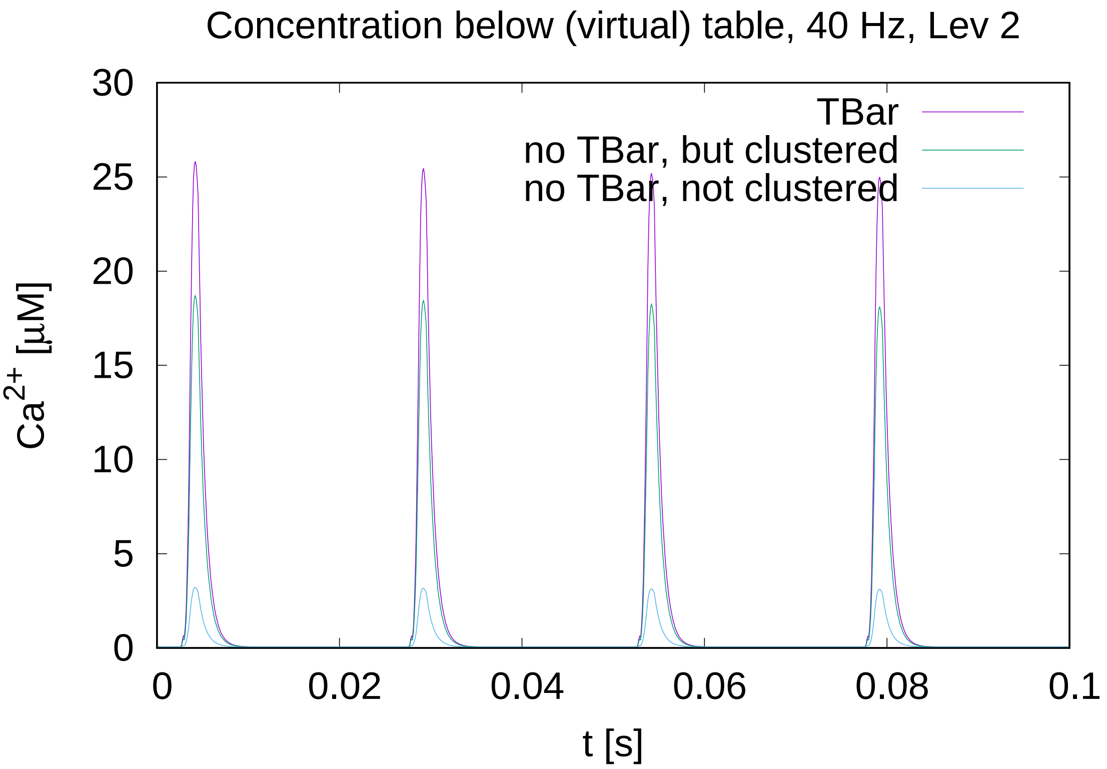

Figure S7. Relative concentrations in computational subdomain $\mathcal{U}$, i.e. the region below the (virtual) table of the TBar. Standard parameter set used.

3 LIST OF ABBREVIATIONS

The following abbreviations are used with in the paper:

EPSP	Evoked Post Synaptic Potential
NMJ	Neuro Muscular Junction
VGCC	voltage gated calcium current
PMCA	Plasma Membrane Calcium ATPase
NCX	Natrium Calcium Exchanger
PDE	Partial Differential Equation
ODE	Ordinary Differential Equation
AZ	active zone
LaMA	Law of Mass Action
DoF	degrees of freedom
